# Cyclic di-GMP cyclase SSFG_02181 from *Streptomyces ghanaensis* ATCC14672 regulates antibiotic biosynthesis and morphological differentiation in streptomycetes

**DOI:** 10.1038/s41598-020-68856-9

**Published:** 2020-07-21

**Authors:** Desirèe Nuzzo, Roman Makitrynskyy, Olga Tsypik, Andreas Bechthold

**Affiliations:** Pharmaceutical Biology and Biotechnology, Institute of Pharmaceutical Sciences, Albert-Ludwigs University, 79104 Freiburg, Germany

**Keywords:** Industrial microbiology, Metabolic engineering, Antimicrobials, Bacteria, Industrial microbiology, Microbial genetics

## Abstract

Streptomycetes are filamentous bacteria famous for their ability to produce a vast majority of clinically important secondary metabolites. Both complex morphogenesis and onset of antibiotic biosynthesis are tightly linked in streptomycetes and require series of specific signals for initiation. Cyclic dimeric 3′–5′ guanosine monophosphate, c-di-GMP, one of the well-known bacterial second messengers, has been recently shown to govern morphogenesis and natural product synthesis in *Streptomyces* by altering the activity of the pleiotropic regulator BldD. Here we report a role of the heme-binding diguanylate cyclase SSFG_02181 from *Streptomyces ghanaensis* in the regulation of the peptidoglycan glycosyltransferase inhibitor moenomycin A biosynthesis. Deletion of *ssfg_02181* reduced the moenomycin A accumulation and led to a precocious sporulation, while the overexpression of the gene blocked sporogenesis and remarkably improved antibiotic titer. We also demonstrate that BldD negatively controls the expression of *ssfg_02181*, which stems from direct binding of BldD to the *ssfg_02181* promoter. Notably, the heterologous expression of *ssfg_02181* in model *Streptomyces* spp. arrested morphological progression at aerial mycelium level and strongly altered the production of secondary metabolites. Altogether, our work underscores the significance of c-di-GMP-mediated signaling in natural product biosynthesis and pointed to extensively applicable approach to increase antibiotic production levels in streptomycetes.

## Introduction

Cyclic dimeric (3′ → 5′) GMP (c-di-GMP) was initially reported by Ross et al.^1^ in 1987 as an allosteric activator of a bacterial cellulose synthase. Since that time, its role has been expanded to control various cellular processes including biofilm formation, planktonic to sessile state transition, cell progression and expression of virulence genes^2^. Alteration of c-di-GMP intracellular levels crucially affect the life cycle of microorganisms. Two classes of enzymes with opposite activities are responsible for the c-di-GMP turnover. Diguanylate cyclases (DGCs) catalyze the synthesis of the second messenger by homodimerization, using two molecules of GTP. Their active site consists of the highly-conservative GG(D/E)EF [Gly-Gly-(Asp/Glu)-Glu-Phe] domain^3,4^. Phosphodiesterases (PDEs) are responsible for c-di-GMP degradation through the catalytic EAL (Glu-Ala-Leu) domain, yielding the linear dinucleotide 5′-phosphoguanylyl-(3′ → 5′)-guanosine (pGpG)^5,6^. Additionally, the less frequent PDEs containing HD-GYP (His-Asp)-(Gly-Tyr-Pro) domain hydrolyze c-di-GMP into two GMP molecules^7^.

Moreover, sensor domains are often associated with catalytic domains, making these proteins responsive to environmental stimuli. PAS (Per-Arnt-Sim) and GAF (mammalian cGMP-regulated PDEs, *Anabaena* adenylyl cyclases and *Escherichia coli* transcription activator FhlA) domains are the most spread among c-di-GMP metabolizing enzymes^2^. Due to their high plasticity, PAS domains are able to bind a large variety of ligands, such as heme, divalent cations and small molecules. In turn, these cofactors empower proteins to be responsive to redox potentials, gaseous ligands and visible light^8^.

Although the influence of c-di-GMP on bacterial lifestyle has been mostly investigated in unicellular, motile bacteria, recent studies pointed out the crucial role of the cyclic dinucleotide in the Gram-positive genus *Streptomyces*^9–15^. Streptomycetes are non-motile, multicellular bacteria, whose life cycle is characterized by a complex morphological differentiation. Starting from a single spore, branches of germ tubes diffuse into the substrate, favoring the growth of vegetative mycelium. In response to stress signals, morphological differentiation is initiated, leading to aerial mycelium development with subsequent formation of spores. Intriguing, the transition from vegetative to aerial mycelium coincides with the starting point for secondary metabolites production^16,17^. Due to its high complexity, *Streptomyces* life cycle is governed by sophisticated regulatory mechanisms, involving numerous global regulators (e.g. Bld and Whi)^17^ as well as several secondary messengers^14,18^. The first evidence that c-di-GMP is involved in morphological development and antibiotic production in streptomycetes appeared in 2010. In *Streptomyces coelicolor*, den Hengst et al.^13^ identified a gene (*cdgA*) encoding a GGDEF-EAL protein. Upon overexpression, the resulting strain displayed a “bald” phenotype (e.g. absence of aerial mycelium) and reduced production of the blue-pigmented antibiotic actinorhodin. Since that time, few more DGC and PDE encoding genes were investigated in *S. coelicolor*. Overproduction of the DGCs CdgB and CdgD resembled the phenotype of *cdgA*-overexpressed mutant strain, where morphological development was blocked at aerial mycelium level^9,10^. Analogous, deletion of two genes encoding for PDEs (*rmdA* and *rmdB*), caused an increase in c-di-GMP levels followed by the appearance of a “bald” phenotype^11^. Recently, studies in *Streptomyces venezuelae* revealed that orthologues of CdgA, CdgB, RmdA, RmdB and a newly identified DGC CdgC control morphological transitions^15^. In 2014, Tschowri et al. described a detailed mechanism regulating morphogenesis in streptomycetes. It was shown that tetrameric form of c-di-GMP mediates the dimerization of the master pleiotropic regulator BldD. Then homodimeric BldD binds to its target promoter sequences, controlling the transcription of developmental-related genes during the vegetative growth^14^. Interestingly, it was also demonstrated in *S. coelicolor* that *cdgA* and *cdgB* are direct targets of BldD^9,13^, revealing another layer of regulation for c-di-GMP metabolizing enzymes. Finally, a recent work published by Gallagher et al.^19^ showed that in *S. venezuelae* a dimer of c-di-GMP is required for the binding between the sporulation-specific σ^WhiG^ factor and its anti-σ (RsiG), leading to the block of differentiation of aerial hyphae into spore chains.

In this study, we focused our attention on *ssfg_02181*, a gene encoding a putative DGC enzyme in *Streptomyces ghanaensis* ATCC14672*.* This strain is famous for the production of a mixture of phosphoglycolipids known as moenomycins^20^. Among them, moenomycin A (MmA) is considered as the founding member of this class of antibiotics. Broad spectrum of activity and its unique mechanism of action make MmA a promising lead against multidrug-resistant pathogenic bacteria (e.g. VRS and MRSA)^21^. In recent years, many studies have been directed to understand the regulatory mechanisms of MmA biosynthesis with the final aim of generating overproducer strains. Structural genes responsible for antibiotic production are located in two *moe* clusters. Uncommonly for streptomycetes biosynthetic gene clusters, no cluster-situated regulators were identified within *moe* clusters^20^. Interestingly, a fine-tuned control over MmA biosynthesis is directly governed by the pleiotropic regulators AdpA, BldA, AbsB^22^ and WblA^23^. AdpA is a well-known transcriptional regulator for morphological differentiation and secondary metabolism in *Streptomyces*^24–26^. The *bldA* gene encodes tRNA^Leu^_UAA_, the only tRNA able to translate the rare UUA codon in GC-rich streptomycetes^27^. WblA is involved in sporogenesis^28^ and was shown to negatively influence MmA production^23^. Additionally, expression of *adpA*, *bldA* and *wblA* is controlled by the master regulator BldD^12,13^. Recently, we showed that manipulation of genes involved in c-di-GMP metabolism in *S. ghanaensis* caused variations of intracellular c-di-GMP levels, thus affecting the binding of BldD to its target promoters. Consequently, MmA production as well as morphogenesis were severely altered^12^.

In this work, we show that the expression of *ssfg_02181* remarkably influences antibiotic biosynthesis and morphological differentiation in *S. ghanaensis*. Deletion of *ssfg_02181* causes a significant decrease of MmA levels and precocious sporulation of the mutant strain. In contrast, overexpression of the gene leads to a substantial increment of antibiotic production and to a morphological arrest at the aerial mycelium stage. In agreement with in silico analysis, we proved that SSFG_02181 effectively acts as a diguanylate cyclase enzyme in vitro and it is able to bind heme. In addition, we showed that transcription of *ssfg_02181* is negatively regulated by BldD. Finally, heterologous expression of *ssfg_02181* in *S. coelicolor* and *Streptomyces albus* displayed a phenotype similar to that gained in *S. ghanaensis*, suggesting that c-di-GMP may play a conservative role among *Streptomyces* spp.

## Results and discussion

### In silico analysis of SSFG_02181, a putative diguanylate cyclase from *S. ghanaensis*

**Figure 1:**
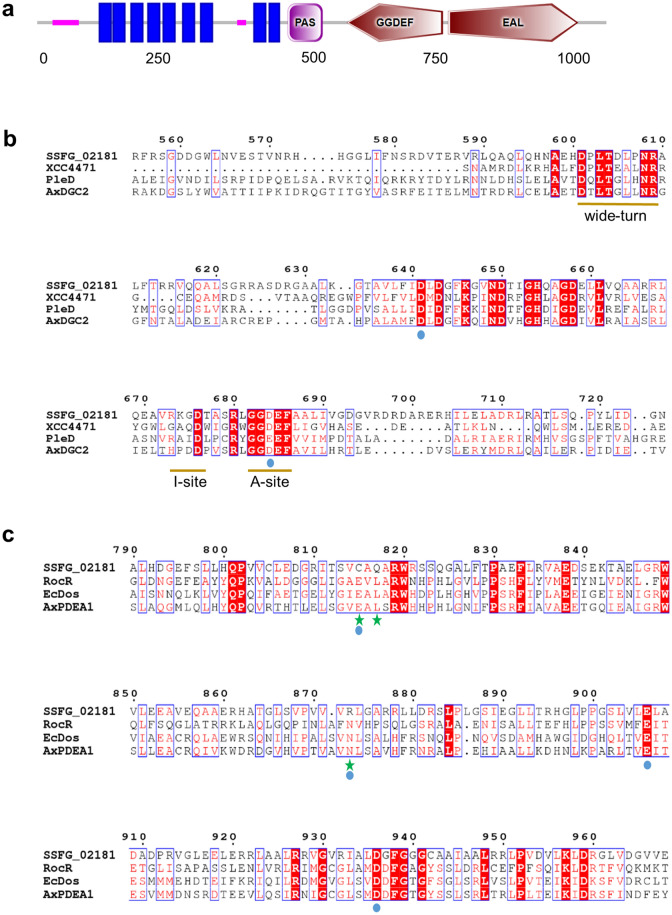
Domains composition of SSFG_02181 and protein sequence alignments. (**a**) Domains architecture of full SSFG_02181 protein predicted by SMART database. Transmembrane-spanning domains are shown as vertical blue bars. (**b**) Sequence alignment of SSFG_02181 GGDEF domain with active GGDEF domains from *Xanthomonas campestris* (XCC4471), *Caulobacter crescentus* (PleD) and *Acetobacter xylinum* (AxDGC2). High conservative residues involved in Mg^2+^ coordination are indicated by blue circles. (**c**) Sequence alignment of degenerated SSFG_02181 EAL domain with the active EAL domains from *Pseudomonas aeruginosa* (RocR), *E. coli* (EcDos) and *Acetobacter xylinum* (AxPDEA1). Green stars indicate mutated key residues in SSFG_02181 EAL domain. The residues are numbered according to the sequence of SSFG_02181. Alignments were performed using Clustal-Omega (https://www.ebi.ac.uk/Tools/msa/clustalo/) and the figures were generated using ESPript 2.2 (https://espript.ibcp.fr/ESPript/ESPript/).

In past years, several studies have marked the importance of the second messenger c-di-GMP in *Streptomyces* life cycle^10,11,13–15^. Interestingly, c-di-GMP metabolizing enzymes were found to be broadly conserved and omnipresent in streptomycetes^15^. Bioinformatic analysis of the *S. ghanaensis* genome identified *ssfg_02181* out of nine genes encoding for putative DGC/PDE enzymes. SSFG_02181 is a 1079-aa protein consisting of a highly-conserved GGDEF domain, followed by a degenerated EAL domain. An N-terminal PAS sensor domain is located upstream the putative DGC active site (A-site), preceded by nine transmembrane-spanning helices (Fig. 1a). Multiple sequences alignment showed SSFG_02181 as a homolog of SCO5511 from *S. coelicolor* and of SVEN_5187 from *S. venezuelae*, sharing 80% (sequence coverage 98%) and 72% (sequence coverage 89%) of identity, respectively.

In order to identify conserved residues crucial for the enzymatic activity in SSFG_02181, Clustal Omega software was used to compare the protein sequence with well-characterized homologous proteins in other bacteria. For this purpose, the functional GG(D/E)EF domains of XCC4471 from *Xanthomonas campestris*^29^, PleD from *Caulobacter crescentus*^3^ and AxDGC2 from *Acetobacter xylinum*^30^ were chosen. As shown in Fig. 1b, SSFG_02181 possesses all five distinctive GGDEF amino acids essential for DGC function. Particularly, two initial glycines are involved in GTP binding, whereas Glu residue at fourth position mediates the ion (Mg^2+^ or Mn^2+^) coordination^3^. Asp (in same cases Glu) at the third position is also involved in metal coordination and is crucial for catalysis^3,31,32^. Additionally, SSFG_02181 GGDEF domain contains the canonical RxxD motif (I-site), responsible for binding of dimeric c-di-GMP and thus mediating its own inhibition. Another feature of an active GGDEF domain is the presence of the invariant DxLT motif, which is required for the formation of a stabilizing “wide-turn” structure and represents the starting point of the catalytic site^33^. Other residues in the vicinity of the active site and required for the activity were also found to be highly conserved in SSFG_02181 GGDEF domain^33^, suggesting that this enzyme functions as DGC (Fig. 1b).

The predicted EAL domain of SSFG_02181 lacks key residues for the PDE activity and Mg^2+^ coordination. In fact, sequence alignment with the enzymatically active PDEs from *E. coli* (EcDos)^34^, *Pseudomonas aeruginosa* (RocR)^35^ and *Acetobacter xylinum* (AxPDEA1)^36^ showed Glu and Leu residues (of the EAL site) substituted with Cys and Gln, respectively (Fig. 1c). Particularly, Glu was proved to have a crucial role in metal coordination and catalysis, mutations of which result in abolishment of the activity^32,37^. Likewise, a second essential residue involved in Mg^2+^ binding (Asn96)^35^ is mutated to Arg in SSFG_02181 EAL sequence, further suggesting that this domain is likely enzymatically inactive. Based on these evidences, we predicted SSFG_02181 to work solely as DGC enzyme.

### SSFG_02181 is an active diguanylate cyclase

**Figure 2:**
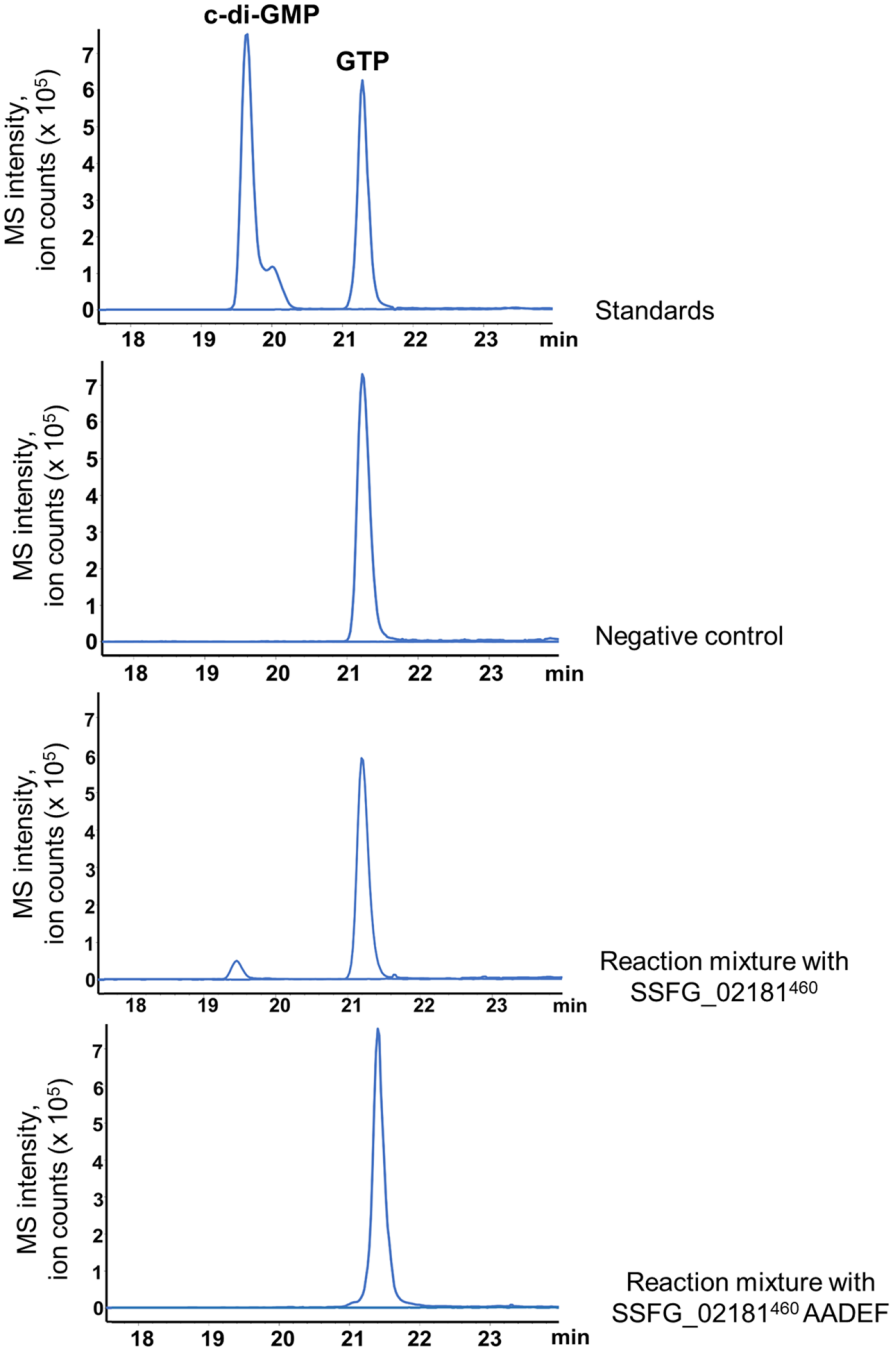
LC–MS detection of c-di-GMP synthesized by SSFG_02181^460^. EIC corresponding to the expected [M-H]^−^ ion of c-di-GMP (*m/z* 689.1) was absent in both negative control mixtures (200 µM GTP, 50 mM Tris-HCl [pH 8.0], 50 mM NaCl and 10 mM MgCl_2_ without native enzyme and with mutated SSFG_02181^460^ AADEF) and distinctly detected in the reaction mixture containing SSFG_02181^460^ (200 µM GTP, 50 mM Tris-HCl [pH 8.0], 50 mM NaCl, 10 mM MgCl_2_ and 5 µM SSFG_02181^460^ protein).

To prove an enzymatic activity of SSFG_02181 in vitro, we heterologously expressed *ssfg_02181* in *E. coli*. Despite all our attempts to gain the full protein, it remained insoluble most likely due to the presence of the nine transmembrane helices. A truncated version of the protein (named SSFG_02181^460^) lacking the first 459 amino acids was purified to homogeneity (Supplementary Fig. S1) from soluble fraction and used for an in vitro assay. Consequently, SSFG_02181^460^ consists of PAS-GGDEF-EAL domains fused with an N-terminal Strep-tag. Strep-tagged SSFG_02181^460^ was incubated at 37 °C for 2 h in the presence of 200 µM GTP, leading to the formation of c-di-GMP. As a control, a reaction mixture without the enzyme was used. Furthermore, a mutated version of Strep-tag-SSFG_02181^460^ (named SSFG_02181^460^ AADEF) carrying both G682A and G683A substitutions in the GGDEF domain was purified and tested for cyclase activity. As shown in Fig. 2, the expected molecular ion of c-di-GMP (*m/z* = 689.1 [M-H]^–^) was detected in the reaction sample but not in the negative controls (see MS spectra in Supplementary Fig. S2).

To examine SSFG_02181 for the PDE activity, we incubated SSFG_02181^460^ with 200 µM of c-di-GMP. As anticipated from in silico analysis, no hydrolysis of c-di-GMP to pGpG was observed (Supplementary Fig. S3). In summary, these results confirmed SSFG_02181 to be an active DGC able to convert GTP to c-di-GMP and showed that mutations in the conservative GGDEF domain result in the loss of its catalytic activity.

### SSFG_02181 is a heme-binding protein

**Figure 3:**
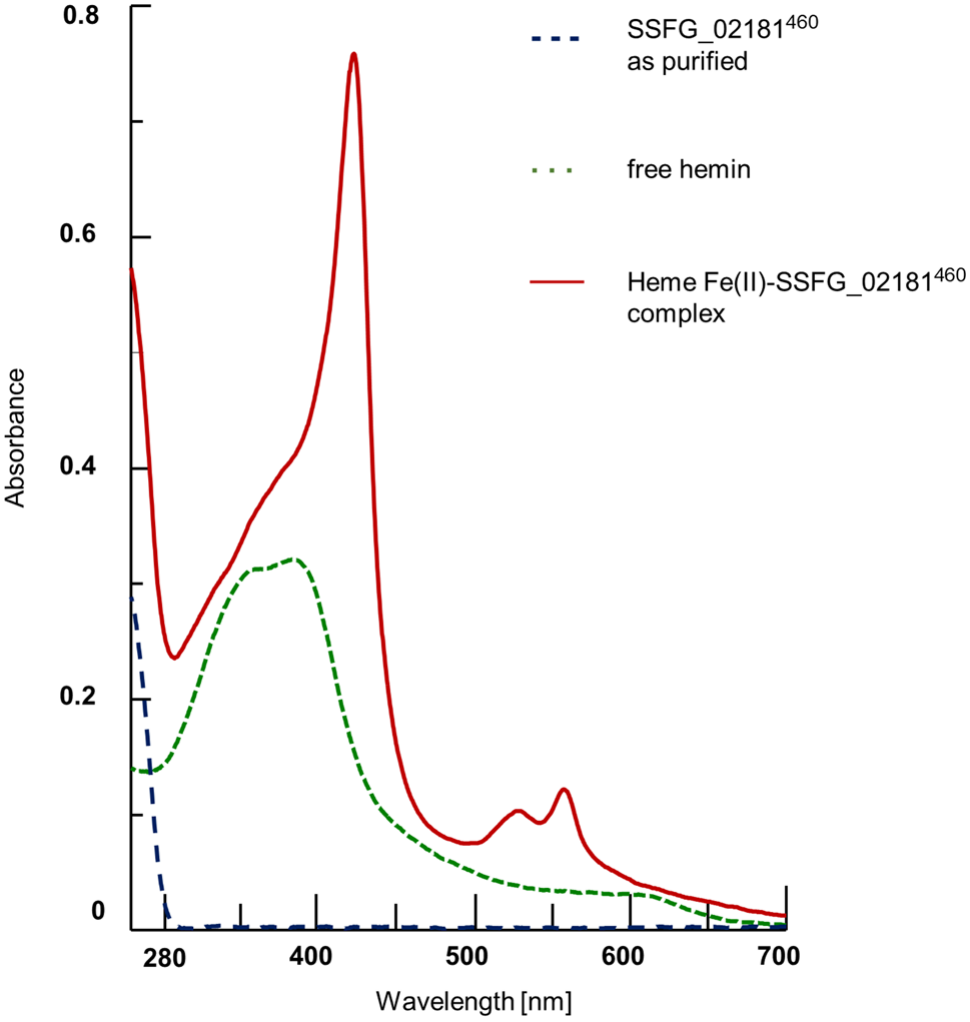
Evaluation of the heme-binding capacity of SSFG_02181^460^ by UV–vis spectroscopy. Absorption spectra of SSFG_02181^460^ as purified (blue dotted trace), hemin (green dotted trace) and heme-SSFG_02181^460^ complex (red solid trace).

c-di-GMP metabolizing enzymes are often associated with sensor domains, such as PAS or GAF^2^. Likewise, a PAS sensor domain is located at the N-terminal sequence of SSFG_02181. Due to their high plasticity, PAS domains are well-known for their ability to adapt to different ligands, such as heme, FAD, divalent metals or even secondary metabolites^8^. Bioinformatic analysis using BLAST software identified a putative heme-binding pocket in the PAS domain of SSFG_02181 (Supplementary Fig. S4). To determine whether SSFG_02181^460^ could effectively bind heme, the protein was mixed with hemin in equimolar amounts (10 µM) and UV–vis absorption spectra were recorded (Fig. 3). As a control, the mixture containing no protein was analyzed. As depicted in Fig. 3, the heme spectrum showed a maximum peak at 384 nm and a shoulder in the 550 to 700 nm region of the spectrum. Conversely, the protein-hemin mixture displayed a clear shift of the Soret band (from 384 to 423 nm) and the sharpening of the α- and β-peaks at 557 nm and 528 nm, respectively. Due to the presence of DTT in the buffer, the obtained spectra represent the Fe^2+^, deoxy form of the free heme and the reduced, deoxy form of the heme-protein complex. Collectively, these results demonstrated that SSFG_02181^460^ is able to bind heme with an equimolar stoichiometry.

Next, we repeated the in vitro assays using either Strep-tagged SSFG_02181^460^ alone or in complex with heme in both Fe(II) and Fe(III) forms. No significant changes in the DGC activity were observed comparing the reaction products of the apoprotein to the heme-protein complexes (Supplementary Fig. S5), suggesting that heme alone is not sufficient to affect the enzymatic activity.

Most likely, the activity of SSFG_02181 might be influenced by sensing gaseous ligands. Several DGC/PDE enzymes have been found to vary their activity upon binding of gases such as O_2_, CO and NO. The DGC activity of the heme-binding protein YddV from *E. coli* is strikingly stimulated when O_2_ is bound to the heme Fe(II) complex, whereas the heme Fe(II) and the heme Fe(II)-NO complexes result inactive^38^. Conversely, the PDEA1 from *Acetobacter xylinum* decreases its enzymatic activity when O_2_ is bound to the protein^36^. To date, just one c-di-GMP metabolizing enzyme in *Streptomyces* was proven to be a hemoprotein. The PDE RmdA from *S. coelicolor* binds hemin through its PAS9 N-terminal domain and it is able to respond to O_2_ and CO, albeit its PDE activity does not change upon the binding^11^. Finally, a truncated version of SSFG_02181 was used for the in vitro assay. In fact, SSFG_02181^460^ lacks nine N-terminal transmembrane domains which might also be involved in signals transduction upon sensing environmental stimuli. Whether the activity of SSFG_02181 is affected by gaseous ligands and/or by inputs of the transmembrane domains remains obscure and requires further investigations.

### Deletion and overexpression of *ssfg_02181* cause impaired morphogenesis and alteration of MmA biosynthesis

**Figure 4:**
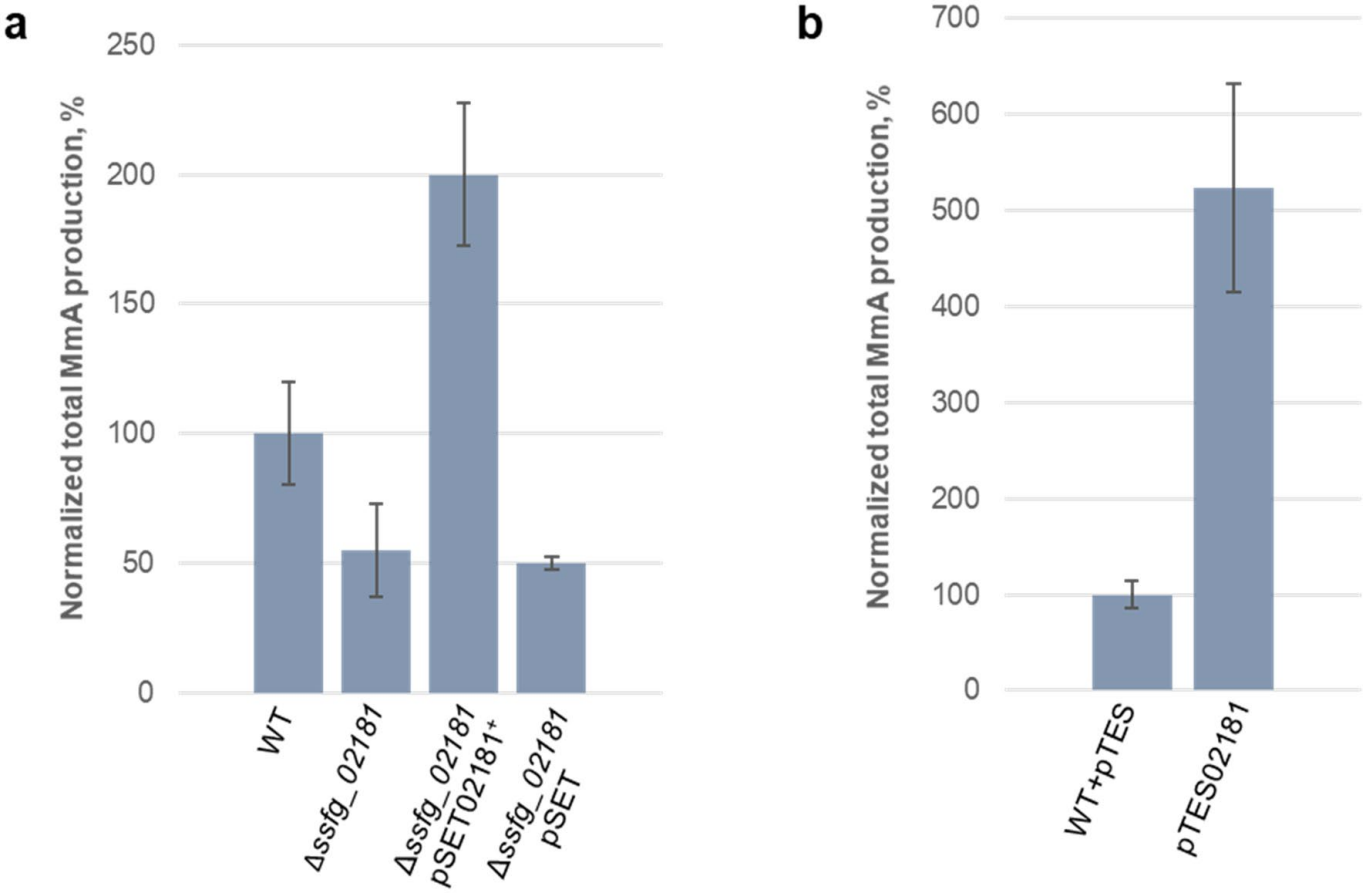
Levels of moenomycin production accumulated in the biomass of different *S. ghanaensis* strains. (**a**) *S. ghanaensis* wild-type (WT), *ssfg_02181* null mutant (Δ*ssfg_02181*), complemented strain (Δ*ssfg_02181* pSET02181^+^) and mutant carrying the empty vector pSET (Δ*ssfg_02181* pSET). (**b**) *S. ghanaensis* wild-type carrying empty pTES vector (WT + pTES) and *ssfg_02181* overexpressed mutant (pTES02181) after 4 days of growth. The mean value of moenomycin mass peak area of *S. ghanaensis* wild-type was taken as 100%. Error bars represent standard deviations.

**Figure 5:**
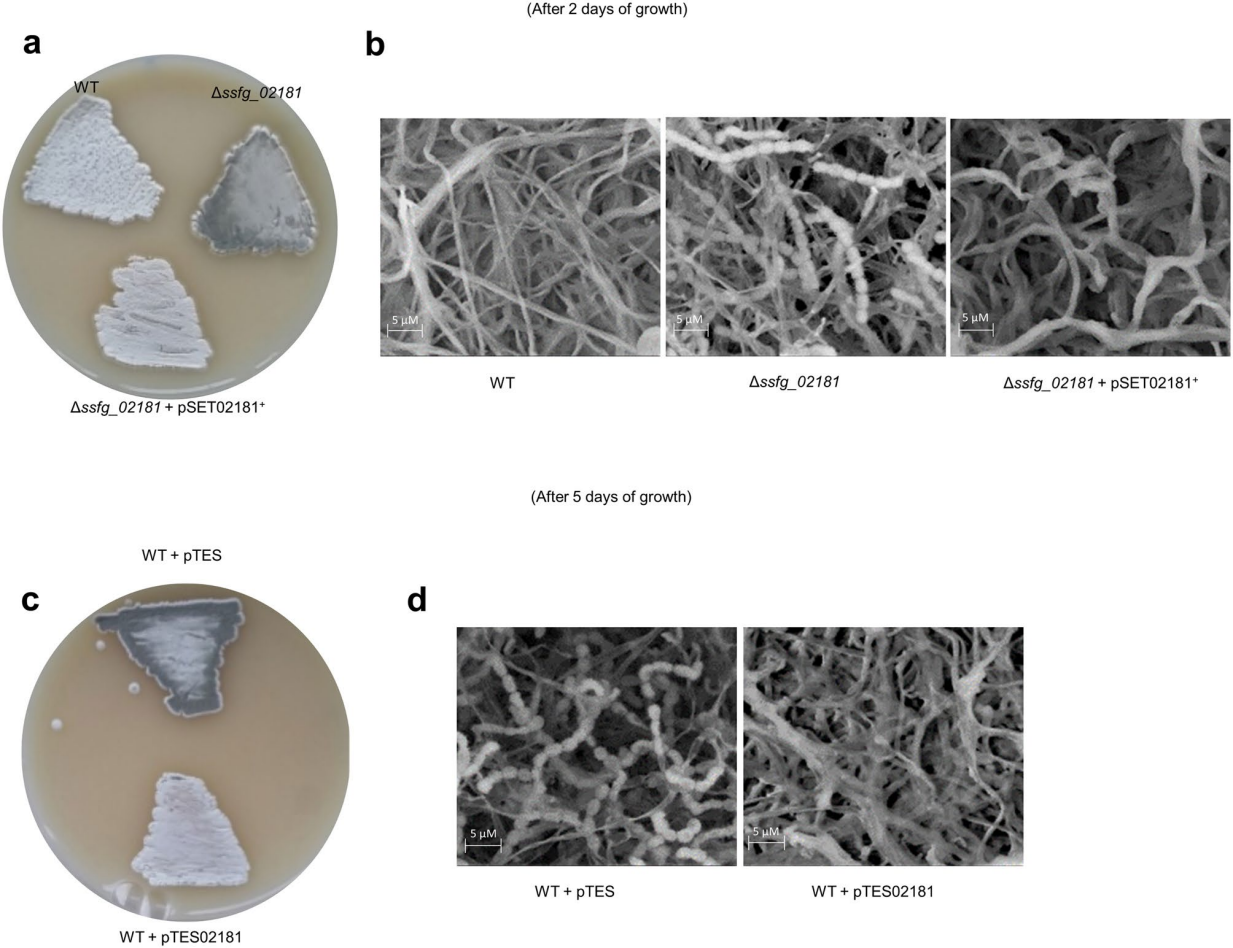
SSFG_02181 activity is crucial for normal morphogenesis. Phenotypes of the *S. ghanaensis* wild-type (WT) compared to *ssfg_02181* overexpressed (pTES02181) and null (Δ*ssfg_02181*) mutants. (**a**) *S. ghanaensis* WT, *S. ghanaensis* Δ*ssfg_02181* and complemented Δ*ssfg_02181* pSET02181^+^ strains grown on SFM agar for 2 days. Disruption of *ssfg_02181* resulted in accelerated sporulation, as revealed by scanning electron microscopy (SEM) (**b**). (**c**) *S. ghanaensis* WT carrying pTES empty vector (WT + pTES) and pTES02181 strains grown on SFM agar for 5 days. Overexpression of *ssfg_02181* resulted in a significant delay in morphological development, as also shown by SEM (**d**).

In order to investigate a role of SSFG_02181 in *S. ghanaensis* development and MmA production, we constructed an *ssfg_02181* marker-free null-mutant through homologous recombination. Analysis of moenomycin production revealed a 2-fold decrease of antibiotic production in Δ*ssfg_02181* in comparison to the wild-type strain (Fig. 4a). In order to exclude polar effect, complementation experiment was performed using the φC31-based integrative vector pSET152. A native copy of the *ssfg_02181* gene, along with its own promoter, was cloned into pSET152 (yielding pSET02181) and integrated into *S. ghanaensis* Δ*ssfg_02181* chromosome. The Δ*ssfg_02181* mutant carrying the empty vector was used as a control. As shown in Fig. 4a, the effects of *ssfg_02181* deletion on MmA production were abolished upon gene complementation. However, a substantial increase of antibiotic accumulation was observed in Δ*ssfg_02181* pSET02181^+^ in comparison to both mutant and wild-type strains. This phenomenon might be due to the integration of an additional copy of the plasmid into pseudo φC31-sites in the genome of *S. ghanaensis* Δ*ssfg_02181*, as it was already shown for others *Streptomyces* spp.^39^. Next, we studied the influence of *ssfg_02181* deletion on morphological development. The mutant strain was cultivated on SFM agar medium for 5 days at 37 °C. After 2 days of growth, Δ*ssfg_02181* displayed the characteristic green pigment associated with spores formation on its surface, whereas the surface of a control strain still remained white (Fig. 5a). Scanning electron microscopy revealed chains of spores in *S. ghanaensis* Δ*ssfg_02181* in contrast to long nonsporulating aerial hyphae of the wild-type strain (Fig. 5b). The wild-type phenotype was restored upon complementation with a native copy of the gene (plasmid pSET02181), implying that the significant decrease of antibiotic production as well as the precocious sporulation were solely due to the *ssfg_02181* disruption.

Further confirmation of the SSFG_02181 involvement in morphogenesis and secondary metabolite production was achieved upon gene overexpression. For this purpose, the *ssfg_02181* coding sequence was placed under control of the strong constitutive promoter *ermEp*, into the φC31-based integrative vector pTES. The resulting pTES02181 plasmid was transferred into *S. ghanaensis*. As a control, the wild-type strain carrying an empty copy of the vector was used. Unlike gene deletion, overexpression of *ssfg_02181* led to 5-fold increase of MmA production (Fig. 4b) and delay in morphological development (Fig. 5c). Scanning electron microscopy revealed that after 5 days the morphological progression of *S. ghanaensis* containing pTES02181 was arrested at the aerial mycelium stage, whereas the control strain, carrying an empty vector, developed mature spores (Fig. 5d).

Our results report the involvement of a c-di-GMP metabolizing enzyme in *S. ghanaensis* life cycle. In a line with our in silico analysis and the enzymatic in vitro assay, we demonstrated that SSFG_02181 is an active DGC. Homologs of SSFG_02181 acting as DGCs have been identified and characterized in other *Streptomyces* spp. In particular, *cdgB* and *cdgC* null-mutants of *S. venezuelae*^15^ exhibit similar phenotype to that obtained after *ssfg_02181* deletion (e.g. precocious sporulation). In addition, overexpression of *ssfg_02181* led to a delay in morphological development, as it was shown for *cdgA*, *cdgB* and *cdgD* overexpressing mutants in *S. coelicolor*^9,10,13^.

Recently, we showed that manipulations of c-di-GMP metabolizing enzymes in *S. ghanaensis* severely affect secondary metabolites production. Indeed, c-di-GMP mediates the dimerization of BldD, which in turn activates *adpA*_*gh*_ expression and inhibits *wblA*_*gh*_ transcription^12^. AdpA was shown to act as a positive transcriptional regulator of key structural genes in *moe* cluster^22^, whereas WblA was shown to negatively regulate MmA production^23^. In this study, deletion of *ssfg_02181* results in a significant decrease of antibiotic production. Similarly to the Δc*dgB*_*gh*_ mutant^12^, we assume that the loss of SSFG_02181 causes a reduction of intracellular c-di-GMP pool and thus the dissociation of BldD dimer from the target promoters (e.g. *adpA* and *wblA*).

In the past years, several studies were conducted to fully understand the functions and mechanisms of action of DGCs and PDEs in streptomycetes. Genetic manipulation and biochemical analysis confirmed that these enzymes are responsible for the metabolism of c-di-GMP, which intracellular level is crucially balanced over the entire bacterial life cycle. Pool of this second messenger in turn regulates both morphogenesis and secondary metabolite production by controlling both BldD and the σ^WhiG^ factor^12,14,19^. However, *Streptomyces* spp. possess more than one c-di-GMP-metabolizing enzymes, suggesting that these proteins might be active at different time points and spatial locations during the cell life. Finally, the majority of these enzymes carry one or more sensor domains whose functions have not yet been fully clarified. Most likely, these domains regulate the catalytic activity by responding to either external or internal stimuli, thus maintaining a well-tuned control over intracellular c-di-GMP levels in the cell.

### Heterologous expression of *ssfg_02181* affects antibiotic production and morphological development in *Streptomyces* spp.

**Figure 6:**
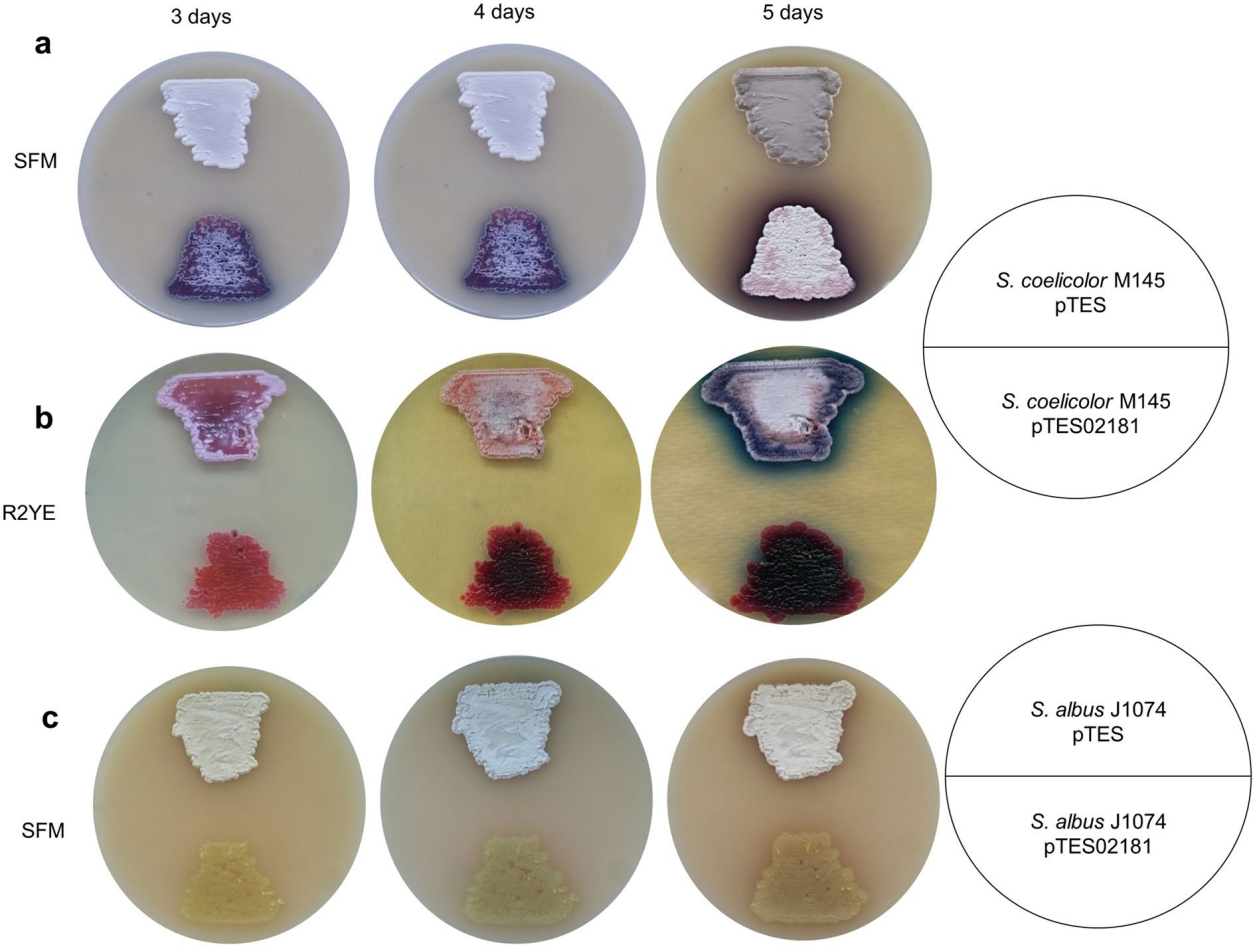
Overexpression of *ssfg_02181* severely affects morphogenesis and secondary metabolite production in *Streptomyces* spp. (**a**, **b**) Phenotypes of *S. coelicolor* M145 + pTES, *S. coelicolor* M145 + pTES02181 and (**c**) *S. albus* J1074 + pTES, *S. albus* J1074 + pTES02181 grown on different agar media.

In order to determine whether overexpression of *ssfg_02181* could affect secondary metabolites production and morphological development in other streptomycetes, we introduced an additional copy of the gene controlled by the strong constitutive promoter *ermEp* (plasmid pTES02181) into two model strains *S. coelicolor* M145 and *S. albus* J1074. As a control, strains carrying an empty copy of the vector pTES were used. After 3 days of growth, *S. coelicolor* pTES02181 exhibited a significant increase of actinorhodin production on SFM agar, in comparison to the control strain (Fig. 6a). Moreover, phenotypic analysis revealed the inability of *S. coelicolor* pTES02181 to develop aerial mycelium on R2YE agar (Fig. 6b) and to sporulate on SFM agar after 5 days of growth (Fig. 6a). Similarly, the overexpression of *ssfg_02181* in *S. albus* J1074 led to an arrest of morphological development on SFM agar in comparison to the control strain (Fig. 6c). These results demonstrate that SSFG_02181 retains its activity upon heterologous expression, suggesting that similar mechanisms of c-di-GMP-mediated regulatory network operate in other streptomycetes. Indeed, protein sequence alignment of SSFG_02181 and its orthologs from *S. coelicolor* (SCO5511), *S. venezuelae* (SVEN_5187), *S. griseus* (SGR_2001) and *S. albus* (XNR_1322) showed that these proteins possess a degenerated EAL domain but share the conservative GGDEF domain (Supplementary Fig. S6), indicating that all of them likely function as DGCs. Due to the fact that orthologs of this gene are broadly conserved in streptomycetes^15^, the overexpression of *ssfg_02181* could be employed as a useful approach to increase antibiotic production titers in other actinobacteria.

### The transcription of *ssfg_02181* is repressed by the master regulator BldD

**Figure 7:**
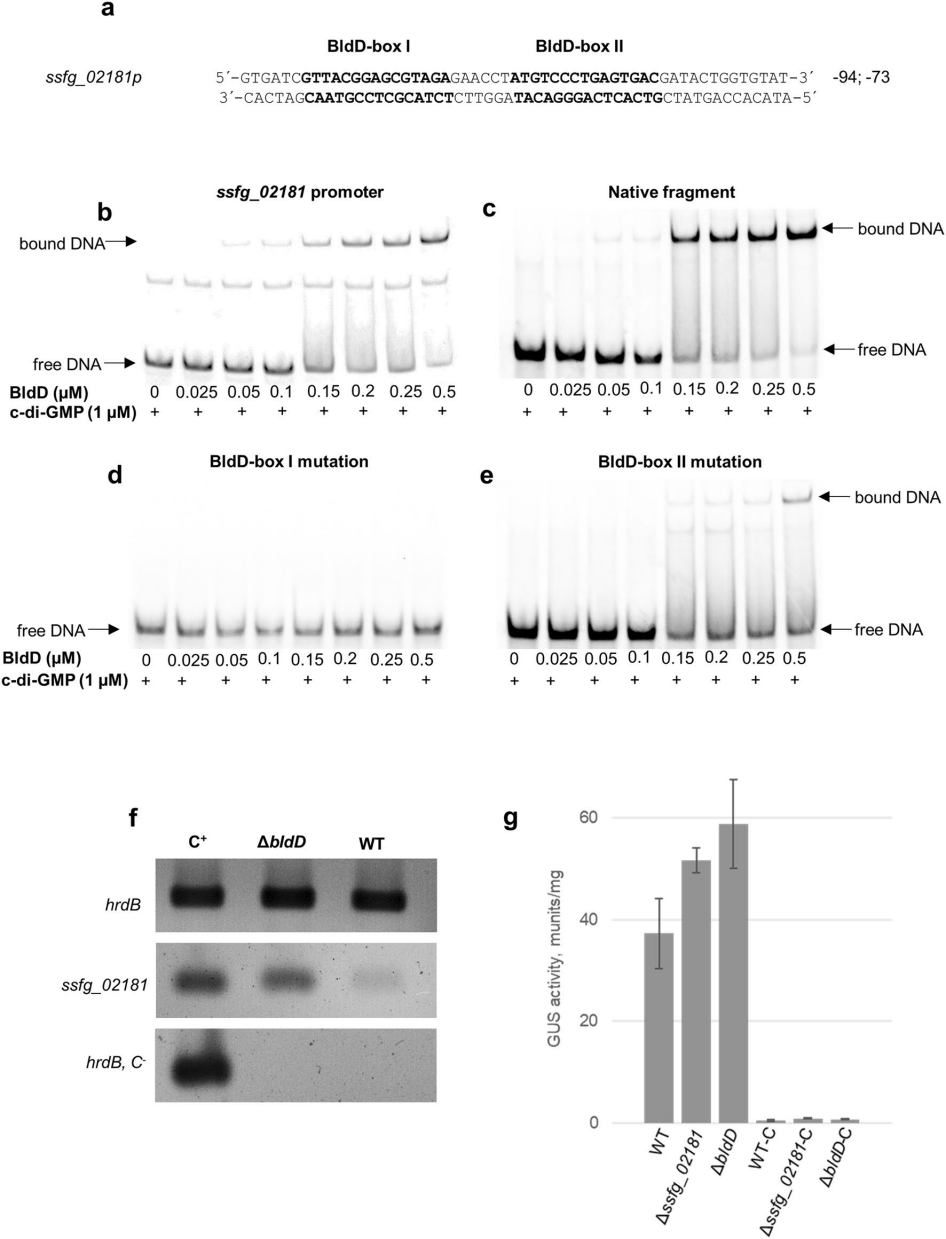
BldD regulates expression of *ssfg_02181*. (**a**) The putative BldD-boxes identified in the promoter of *ssfg_02181* are marked in bold. Numbers represent the distance from the putative start codon of the downstream gene. (**b**) EMSA analysis of BldD binding to the promoter region of *ssfg_02181*. (**c**) EMSA analysis of BldD binding to a DNA fragment carrying native BldD-boxes sequences. (**d**) EMSA analysis of BldD binding to a DNA fragment carrying a mutation in BldD-box I. (**e**) EMSA analysis of BldD binding to a DNA fragment carrying a mutation in BldD-box II. (**f**) Comparison of transcriptional profiles of *ssfg_02181* in the *S. ghananesis* wild-type (WT) and Δ*bldD* strains. The expression of tested gene was analyzed in 48 h cultures grown in TSB; 200 ng of cDNA were used per reaction. Total RNA samples were isolated from three independent biological replicates. The images represent the typical result of three independent RT-PCR experiments and derive from cropped sections of original agarose gels electrophoresis. Unprocessed, full-length gels are presented in Supplementary Fig. S9. C^+^ (positive control) corresponds to the genomic DNA of *S. ghanaensis* WT strain. C^−^ (negative control) represents the attempts to synthesize *hrdB* from RNA without pretreatment with Reverse transcriptase. (**g**) Transcriptional activity of the *ssfg_02181* promoter in different *S. ghanaensis* strains. WT-C, Δ*ssfg_02181*-C and Δ*bldD*-C carry the empty pGUS vector. Cultures and subsequent β-glucuronidase measurements were done in triplicate. Values were normalized to equal amounts of dry biomass. Error bars represent standard deviations.

The master pleiotropic regulator BldD is one of the most conserved transcriptional factors in streptomycetes, controlling diverse processes in the cell^13,40^. Recently, den Hengst et al.^13^ identified the BldD-binding consensus site, consisting of the 15-bp palindromic sequence nTnACnC(A/T)GnGTnAn, named BldD-box. In *S. coelicolor*, BldD-box was found to be located also in the promoter regions of three genes encoding for c-di-GMP metabolizing enzymes (*cdgA*, *cdgB* and *sco5511*)^13^. Likewise, bioinformatic analysis^12^ on *ssfg_02181* promoter region identified two sequences (TCTACGCTCCGTAAC and ATGTCCCTGAGTGAC) (Fig. 7a) resembling the consensus BldD-box. These sequences showed high similarity to the ones uncovered by den Hengst et al.^13^ in *sco5511* from *S. coelicolor*.

Alignment of BldD from *S. ghanaensis* and its orthologs from *S. coelicolor*, *S. venezuelae* and *S. albus* revealed an identical DNA binding domain (DBD) and the presence of both c-di-GMP binding motifs RxD-X_8_-RxxD (Supplementary Fig. S7a), showing that BldD in *S. ghanaensis* retains all the features required to function as a transcriptional regulator. Indeed, BldD was recently proved to bind to the promoter sequence of *cdgB*_*gh*_ in *S. ghanaensis*^12^.

To determine BldD binding affinity to the *ssfg_02181* promoter region, we performed an electrophoretic mobility shift assay (EMSA). An N-terminally His_6_-tagged BldD protein was produced in *E. coli* and purified. Increasing amounts of BldD were incubated with the ^33^P-radiolabeled promoter region of *ssfg_02181* at the presence of 1 µM c-di-GMP. As shown in Fig. 7b, BldD was able to bind the target sequence at concentration starting from 0.05 µM. Increasing concentrations of BldD strongly increased binding efficacy, resulted in an almost complete DNA shift at 0.5 µM. To prove the specificity of the BldD binding, excess of unlabeled *ssfg_02181*-promoter DNA was used to compete with the radiolabeled one for BldD (Supplementary Fig. S8a). Moreover, we confirmed that the affinity between BldD and *ssfg_02181* promoter is affected by the presence of c-di-GMP. In fact, increasing concentration of c-di-GMP greatly enhanced BldD binding to the target promoter region, whereas the elimination of the second messenger from the reaction mixture resulted in no DNA shift (Supplementary Fig. S8b).

Next, we aimed to investigate which of two identified consensus sequences TCTACGCTCCGTAAC (here named BldD-box I) and ATGTCCCTGAGTGAC (here named BldD-box II) effectively corresponds to the major BldD-box in the *ssfg_02181* promoter region. To do this, we designed a set of oligonucleotide sequences. One of them carried a nonsense mutation in the BldD-box I, another in the BldD-box II and the last in both simultaneously. As a control, a nucleotide sequence carrying both native BldD-boxes was used. From the results of EMSA, depicted in Fig. 7c, BldD could bind to the native oligonucleotide fragment already at concentration of 0.05 µM, whereas the mutation in BldD-box I completely inhibited the binding (Fig. 7d). Conversely, the mutation in BldD-box II caused an alteration of the binding affinity, resulting in a higher concentration of BldD required for the binding (0.15 µM) and less amount of shifted DNA (Fig. 7e). Finally, the lack of both sites led to analogous effects observed with the mutation of BldD-box I (Supplementary Fig. S8c).

Next, we analyzed the influence of BldD on the transcription of *ssfg_02181* by semiquantitative (sq)RT-PCR and GusA reporter assay^41^. For sqRT-PCR, the cDNA from *S. ghanaensis* wild-type and *S. ghanaensis* Δ*bldD*^12^ were used as template. As depicted in Fig. 7f, the transcriptional levels of *ssfg_02181* were found to increase in *S. ghanaensis* Δ*bldD*, in comparison to the wild-type strain. This observation was further confirmed by the GusA reporter system, showing a 1.7-fold increase in the transcriptional activity of the *ssfg_02181* promoter in the *bldD* mutant (Fig. 7g). Likewise, deletion of *ssfg_02181* in *S. ghanaensis* caused an increase in the transcriptional activity of its own promoter (Fig. 7g). We propose that the lack of SSFG_02181 causes a drop in c-di-GMP pool, which favors the dissociation of BldD dimer from the *ssfg_02181* promoter. Taken together, these data confirmed that BldD effectively binds to the promoter of *ssfg_02181* and represses its transcription. Based on our results, we could demonstrate that BldD-box I represents the primary binding site of BldD on *ssfg_02181* promoter. Furthermore, we showed that BldD-box II is likely to be required for enhancing the binding efficacy between BldD and *ssfg_02181* promoter. These consensus sites were also identified in the promoter of *sco5511* in *S. coelicolor*^13^ and multiple sequence alignment revealed that both BldD-boxes are shared and highly-similar in *ssfg_02181* promoter and its orthologs (Supplementary Fig. S7b). We speculate that the presence of BldD-box II might reinforce the binding affinity of BldD to the target promoter, regulating the *ssfg_02181* transcription at different BldD/c-di-GMP concentrations. Nevertheless, the biological significance of BldD-box II in vivo remains obscure and needs to be investigated in the future.

In addition, we hypothesize that intracellular c-di-GMP levels undergo a fine-tuned regulation through a negative feedback mechanism. Notably, the expression of the DGC-encoding genes (e.g. *cdgA*, *cdgB* and orthologs of *ssfg_02181*) is under control of BldD. These genes were found to be omnipresent among streptomycetes^15^, indicating that such mechanism might be universally present across *Streptomyces*. Likely, the transcription of these genes is blocked by the BldD dimer when c-di-GMP concentrations reach a certain threshold. As result, the homeostasis of the second messenger is retained during the entire bacterial life cycle. However, it can not be excluded that the binding of BldD might be also required to temporally control the transcription of these genes. Studies in *S. venezuelae* revealed that *cdgB* is expressed in all developmental stages, whereas expression of *cdgC* (ortholog of *ssfg_02181*) increases over the time and reaches the highest levels during sporulation. Finally, *cdgA* is the least transcribed among all DGCs^15^. Therefore, all these findings point to the conclusion that regulatory mechanisms governing the c-di-GMP levels are immensely intricate and well-tuned to permit a coordinate morphological progression and antibiotic synthesis in actinobacteria.

## Conclusions

In the past few years, c-di-GMP has emerged as a crucial molecule involved in streptomycetes development, controlling both morphogenesis and secondary metabolite production^9–12,14,15^. The intracellular c-di-GMP turnover is modulated by the coordinated activities of DGCs and PDEs, encoded by streptomycetes genome. In this study, we demonstrated that the DGC SSFG_02181 affects MmA biosynthesis and morphogenesis in *S. ghanaensis*. Recently, two more c-di-GMP metabolizing enzymes in *S. ghanaensis*, CdgB_gh_ and RmdB_gh_, were found to be involved in secondary metabolite production and morphological development^12^. Altogether, these data demonstrate the decisive role of c-di-GMP in *S. ghanaensis* life cycle. Additionally, we found that the transcription of *ssfg_02181* is directly controlled by the pleiotropic regulator BldD, providing an evidence that the intracellular c-di-GMP pool is strictly regulated to maintain its homeostasis during the bacterial life cycle. Furthermore, we showed that heterologous expression of *ssfg_02181* affects antibiotic production and morphological development in model *Streptomyces* spp. Finally, we found that SSFG_02181 is able to bind heme, albeit the correlation between the enzymatic activity and the ligand binding remains to be further investigated. Collectively, our study provides an effective approach to improve the production of secondary metabolites in various actinobacteria, coupling genetic manipulation of c-di-GMP metabolizing enzymes to their functional analysis.

## Materials and methods

### Strains, plasmids and growth conditions

The bacteria strains and plasmids used in this work are listed in Supplementary Table S1. Luria–Bertani (LB) medium was used to grow *E. coli* strains. *S. ghanaensis* strains were grown on soya flour mannitol agar (SFM) and oatmeal agar media and in TSB liquid medium at 37 °C. *S. coelicolor* M145 was grown on SFM and R2YE agar media at 28 °C. *S. albus* J1074 was cultivated on SFM at 28 °C. Where necessary, appropriate antibiotics were added to the media.

### DNA manipulation and intergeneric conjugation

Polymerase chain reactions (PCRs) were performed using Phusion polymerase (ThermoFisher). All primers used in this work are listed in Supplementary Table S2. Plasmid/chromosomal DNA isolations were carried out using standard procedures^42^. All plasmids were verified by enzymatic digestion, PCR or sequencing. Conjugal transfer of plasmids from *E. coli* to *Streptomyces* spp. was achieved using the *dam dcm* mutant strain *E. coli* ET12567, carrying the helper plasmid pUZ8002 and performed as described previously^43^.

### Scanning electron microscopy

For morphological evaluation, a slice of *S. ghanaensis* strains was recovered from the SFM agar plates and placed on a metal stub. Each sample was introduced into a Quanta 250 environmental scanning electron microscope (SEM) and images were captured at magnification 12,800 ×.

### Bioinformatic analysis

SSFG_02181 protein sequence was analyzed by the SMART domain database (https://smart.embl-heidelberg.de/). Multiple sequence alignments of BldD and the DGC and PDE domains were constructed using the Clustal Omega software (https://www.ebi.ac.uk/Tools/msa/clustalo/) and the figures were generated using the ESPript 2.2 software (https://espript.ibcp.fr/ESPript/ESPript/). BLAST software was employed to identify a putative heme-binding site in SSFG_02181 sequence. Finally, the identification of a BldD-binding site on *ssfg_02181* promoter was done as described elsewhere^12^ and the alignment of *ssfg_02181* promoter and its orthologs was performed using Clustal Omega software.

### Construction of *S. ghanaensis ssfg_02181* null mutant and plasmid for complementation experiment

The DNA fragment containing *ssfg_02181* coding sequence and its flanking regions was amplified from the genomic DNA of *S. ghanaensis* by PCR (primers 02181_del_for and 02181_del_rev) and cloned into the EcoRV-digested pBluescriptKS vector, yielding pBlue02181. The apramycin resistance cassette (*aac(3)IV*), flanked by *loxP* sites, was amplified from pLERECJ using 02181_kn_for and 02181_kn_rev primers. pBlue02181 and *aac(3)IV* were introduced into *E. coli* BW25113/pIJ790 and *ssfg_02181* was replaced with the gene marker by REDIRECT technology^44^. The resulting fragment *02181::aac(3)IV* was amplified by PCR using the same primer pair and cloned into the suicide EcoRV-digested pKGLP2 vector (hygromycin resistance), yielding pKG02181::aac(3)IV. The latter was transferred into *S. ghanaensis* by conjugation. Double-crossover mutants were selected for apramycin resistance and hygromycin sensitivity. The replacement of *ssfg_02181* with the *aac(3)IV* gene marker in the genome of *S. ghanaensis Δ02181::aac(3)IV* was confirmed by PCR. In order to excise the apramycin cassette and yield a marker-free mutant, the Cre-expressing helper plasmid pUWLCre was introduced into *S. ghanaensis* Δ*02181::aac(3)IV* by conjugation^45^. The exconjugants were selected for resistance to thiostrepton followed by screening for sensitivity to apramycin, yielding *S. ghanaensis* Δ*ssfg_02181*. The deletion of apramycin cassette from the genome of *S. ghanaensis* Δ*ssfg_02181* was confirmed by PCR.

For complementation experiment, the *ssfg_02181* coding sequence, along with its own promoter region, was amplified by PCR (primers 02181_compl_for and 02181_compl_rev) and digested with XbaI and EcoRI. The fragment was cloned into the XbaI-EcoRI digested pSET152 vector, to gain pSET02181. The latter was then transferred into *S. ghanaensis* Δ*ssfg_02181* by conjugation.

### Overexpression of *ssfg_02181* in *S. ghanaensis*, *S. coelicolor* and *S. albus*

In order to create the *ssfg_02181*-overexpressed *S. ghanaensis* strain, the DNA fragment comprising only the coding sequence of *ssfg_02181* was amplified by PCR (primers 02181_exp_for and 02181_exp_rev) and digested with XbaI and EcoRI. Next, the resulting fragment was cloned under the control of the strong, constitutive promoter *ermEp* in the integrative pTES vector, linearized by XbaI and EcoRI. The resulting plasmid pTES02181 was transferred into *S. ghanaensis*, *S. coelicolor* M145 and *S. albus* J1074 strains by conjugation.

### Analysis of moenomycin production

The quantification of moenomycin production levels was done as described previously^46,47^. Briefly, *S. ghanaensis* strains were grown in 50 ml TSB medium for 4 days, in triplicate. The cellular pellet was collected by centrifugation and mixed with 10 ml of methanol overnight. The resulting extracts were concentrated in vacuo and dissolved in methanol prior analysis by HPLC–MS. The analysis was performed as established previously^12^. In this work, moenomycin refers to the mixture of the following main compounds: MmA ([M-H]^−^ = 1,580.6 Da) and the precursor nosokomycin B (NoB; [M-H]^−^ = 1,484.6 Da). The experiments were repeated at least three times to ensure reproducibility of results and the levels of antibiotic were referred back to the equal amount of dry biomass (10 mg) in different strains. The data shown in Fig. 2 represent the mean values of independent experiments. The mean value of moenomycin mass peak area of *S. ghanaensis* wild-type was taken as 100%. Error bars indicate the standard deviations.

### Proteins production and purification

The truncated N-terminal Strep-tagged version of SSFG_02181 was produced as following. DNA sequence encoding the PAS-GGDEF-EAL domains of SSFG_02181 (here named SSFG_02181^460^) was amplified by PCR (primers 02181_460_for and 02181_460_rev) and digested with BamHI and HindIII. The digested fragment was cloned into BamHI-HindIII linearized pET51b vector, yielding pET02181^460^. To generate a mutated version of SSFG_02181^460^ (named SSFG_02181^460^ AADEF), PCR mutagenesis was applied to amplify a 7 kb DNA fragment from pET02181^460^ using primers designed to bring G682A and G683A substitutions (02181_460_aadef_for and 02181_460_aadef_rev). The resulting amplicon was treated with T4 Polynucleotide kinase and then self-ligated, yielding pET02181^460^_AADEF. pET02181^460^ and pET02181^460^_AADEF were individually introduced into *E. coli* BL21 Star (DE3) pLysS. The strain was grown at 37 °C to OD_600_ of 0.5 and the protein production was induced with 0.2 mM IPTG. Following 5 h of incubation at 22 °C, cells were harvested by centrifugation and the pellet was resuspended in Step-tag equilibration buffer (50 mM Tris-HCl, 0.5 M NaCl, 1 mM MgCl_2_, 5 mM β-mercaptoethanol and 5% glycerol, pH 8.0). Cell lysis was achieved using three passages through French-press and the lysate was centrifuged at 14.000 rpm for 35 min at 4 °C. The soluble cell extract was loaded onto a column containing 4 ml of pre-equilibrated Strep-Tactin resin (IBA). Fractions were eluted using Strep-tag equilibration buffer containing 2.5 mM desthiobiotin and pooled together. Size-exclusion chromatografy was performed using the ÄKTA fast protein liquid chromatography (FPLC) system, equipped with a Superdex 200 h 16/60 column. After gel filtration, the protein was stored at − 80 °C in a buffer containing 50 mM Tris-HCl, 0.5 M NaCl, 1 mM MgCl_2_, 1 mM dithiotreitol and 5% glycerol, pH 7.5.

### Enzymatic in vitro assay to determine diguanylate cyclase activity

Enzymatic in vitro activity assay was performed by adding 5 µM Strep-tagged SSFG_02181^460^ protein into a reaction mixture (total volume 100 µl) containing 200 µM GTP, 50 mM Tris-HCl [pH 8.0], 50 mM NaCl and 10 mM MgCl_2_. The mixture was incubated at 37 °C for 2 h. The reaction was stopped by adding 10 mM CaCl_2_, followed by incubation at 70 °C for 5 min. LC–MS was used to analyze the reaction products, as described previously^12^. C-di-GMP and GTP standards were purchased from InvivoGen and Sigma-Aldrich, respectively.

### Heme reconstitution and UV–vis spectroscopy

In order to determine whether Strep-tagged SSFG_02181^460^ protein could bind heme, hemin (stock solution 1 mM in DMSO) was added to the purified protein (10 µM) at equimolar concentrations. Following a 30 min incubation at room temperature, the UV–vis absorption spectra were measured with 100 µl protein-hemin complex using a Jasco V-650 spectrophotometer. A solution of 10 µM hemin in a protein-storage buffer was prepared as a control. All experiments were performed under anaerobic conditions in a glove-box at room temperature.

### Electrophoretic mobility shift assay

The DNA fragment corresponding to the promoter region of *ssfg_02181* was amplified by PCR using primers 02181_EMSA_for and 02181_EMSA_rev and then 5′-end labelled with [γ-^33^P] using T4 polynucleotide kinase. 20 fmol of labelled DNA was incubated with increasing concentrations of His6-tagged BldD^12^ in 15 µl buffer (10 mM Tris [pH 7.5], 1 mM EDTA, 5% glycerol, 10 mM NaCl and 1 mM MgCl_2_) containing 1 µg poly(dI-dC) and 1 µM c-di-GMP. The reaction products were separated on 8% native polyacrylamide gel in TBE buffer. The bands were visualized by phosphorimaging. Competition assay was performed in reaction sample containing 20 fmol labelled *ssfg_02181* promoter incubated with 0.5 µM BldD, 1 µM c-di-GMP and 10-, 50-, 100- and 250-fold molar excess of unlabeled probe in binding buffer, as described above. Finally, to evaluate whether c-di-GMP might affect the binding efficiency between BldD and *ssfg_02181* promoter, the latter (20 fmol) was labelled and incubated with 0.1 µM BldD and increasing concentrations of c-di-GMP (0, 0.25, 0.5, 1, 1.5, 2, 2.5, 3 and 4 µM).

### Mutagenesis of BldD-binding sites on *ssfg_02181* promoter

In order to identify the major BldD binding site in *ssfg_02181* promoter region, three pairs of 53 bp complementary oligonuleotide strands were designed where BldD-boxes were replaced by a poly(A) non-sense sequence. Particularly, PmutI_for and PmutI_rev primers were used for mutating BldD-box I, PmutII_for and PmutII_rev for mutating BldD-box II and PmutIII_for and PmutIII_rev for mutating both boxes simultaneously. As a control, a pair of complementary oligonuclotide strands (Pnat_for and Pnat_rev) carrying both native sequences was used. Complementary oligonucleotides were mixed together at a 1:1 molar ratio and diluted in a buffer (10 mM Tris [pH 7.5], 100 mM NaCl and 1 mM EDTA) to a final concentration 1 pmol/µl. To anneal, the tube with oligonucleotides was incubated in a boiling water for 2 min and let slowly cool down to room temperature. Hybridized oligonucleotides were then 5′-end labelled with [γ-^33^P]-ATP using T4 polynucleotide kinase and applied for electrophoretic mobility shift assay experiments.

### β-glucuronidase activity measurements

To investigate the transcriptional activity of *ssfg_02181* promoter, the DNA fragment containing the promoter region of *ssfg_02181* was amplified by PCR (primers 02181_script_for and 02181_script_rev) and digested with XbaI and KpnI. The obtained fragment was cloned into XbaI, KpnI-linearized pGUS vector, yielding pGUS02181. The latter was transferred into *S. ghanaensis* strains by conjugation. β-glucuronidase activity was measured as described previously^41^. The experiments were carried out at least three times. The values refer back to equal amount of dry biomass (10 mg) and correspond to the mean of independent experiments. Error bars indicate the standard deviations.

### Semiquantitative (sq)RT-PCR

Semiquantitative RT-PCR was used to determine differences in the transcription levels of *ssfg_02181* between *S. ghanaensis* strains. sqRT-PCR experiments were performed as described previously^12^. Briefly, total RNA samples were isolated in triplicate from streptomycetes cultures grown in TSB for 48 h. One mcg of total RNA per reaction was used to synthesize cDNA using Photoscript II Reverse Transcriptase (NEB). Two hundred ng of cDNA were used for PCR (primers 02181_check_for and 02181_RT_rev) and the expression levels of the gene were estimated by visual examination. As a control, primers specific to the sequence of *hrdB* encoding the RNA polymerase principal sigma factor were used^12^.

## Supplementary information

Supplementary Information.
