# Supplementary material for: Cyclic di-GMP cyclase SSFG_02181 from Streptomyces ghanaensis ATCC14672 regulates antibiotic biosynthesis and morphological differentiation in streptomycetes
Source: Sci Rep. 2020 Jul 21;10:12021. doi: 10.1038/s41598-020-68856-9 (PMC7374567; doi:10.1038/s41598-020-68856-9)
Supplement: Supplementary file 1 — Supplementary Information. [file 41598_2020_68856_MOESM1_ESM.pdf]

Supplementary information

Cyclic di-GMP cyclase SSFG_02181 from *Streptomyces ghanaensis* ATCC14672 regulates antibiotic biosynthesis and morphological differentiation in streptomycetes

Desirèe Nuzzo[1], Roman Makitrynskyy[1*], Olga Tsypik[1] & Andreas Bechthold[1*]

[1]Pharmaceutical Biology and Biotechnology, Institute of Pharmaceutical Sciences, Albert-Ludwigs University, Freiburg 79104, Germany
Correspondence and requests for materials should be addressed to R.M. (email: roman.makitrynskyy@pharmazie.uni-freiburg.de) or A.B. (email: andreas.bechthold@pharmazie.uni-freiburg.de)

Supplementary Table S1. Strains and plasmids used in this work.

Strains or plasmids	Description/Functions	Source
Strains		
S. ghanaensis ATCC14672	Wild type (WT) moenomycin producer	ATCC
S. ghanaensis Δ*ssfg_02181*	WT derivative, Δ*ssfg_02181* deletion	This work
S. ghanaensis pTES02181	WTderivative, *ssfg_02181* overexpression	This work
S. ghanaensis Δ*bldD*	WT derivative, Δ*bldD* deletion	[1]
S. coelicolor M145	Model organism; heterologous host	[2]
S. albus J1074	Heterologous host	[3]
E. coli XL1Blue	Host strain for DNA cloning	Agilent
E. coli ET12567 (pUZ8002)	Host for *E. coli*-streptomycetes conjugation	[4]
E. coli BW25113	Host for REDIRECT technology with helper plasmid pIJ790	[5]
E. coli BL21 Star (DE3)/pLysS	Host for protein production	Thermo Fisher Scientific
Plasmids		
pBluesctiptIIKS+	Cloning vector, Ap^{R}	Addgene
pKGLP2	Suicide vector carrying *gusA*, Hyg^{R}	[6]
pSET152	φC31-based integrative vector, Am^{R}	[7]
pUWLCre	Vector carrying *cre* under *ermEp*, Tsr^{R}	[8]
pTES	pSET152 derivative carrying *ermEp*, Am^{R}	[9]
pLERECJ	Vector carrying apramycin resistance cassette with loxP-sites for gene replacement	Prof. Luzhetskyy, Saarland University
pET51b	Vector for protein production, Ap^{R}	Novagen
pGUS	pSET152 derivative carrying reporter gene *gusA*, Am^{R} Sp^{R}	[6]
pBlue02181	pBluescriptIIKS+ carrying *ssfg_02181*	This work
pBlue02181::aac(3)IV	pBlue02181, Δ*ssfg_02181* deletion	This work
pSET02181_compl	pSET152 carrying *ssfg_02181* along with its promoter	This work
pKG02181::aac(3)IV	pKGLP2 carrying *02181::aac(3)IV*	This work
pTES02181	pTES carrying *ermEp-ssfg_02181* fusion	This work
pET02181^{460}	pET51b carrying Strep-tagged SSFG_02181^{460}	This work
pET02181^{460} AADEF	pET51b carrying a mutated version of Strep-tagged SSFG_02181^{460}	This work
pGUS02181_script	pGUS carrying *ssfg_02181* promoter-*gusA* fusion	This work

Ap^{R} (ampicillin resistance); Am^{R} (apramycin resistance); Hyg^{R} (hygromycin resistance); Tsr^{R} (tiostreptone resistance); Km^{R} (kanamycin resistance); Sp^{R} (spectinomycin resistance)

Supplementary Table S2. Primers used in this work.

Primers	Sequence
02181_del_for	AAATCTAGACATCATCTCGGCCGTGAA
02181_del_rev	AAAGAATTCATGGAGTTCGCCTCCTCGA
02181_kn_for	CGCTGACCTACGGCGCCGGGGACGGACAGTGCGGATGGACTGGATATCTCTAGATACCG
02181_kn_rev	CGCATGGAACTGACCCTCCTCCCGGCACACCCTCACTGTCAAACAAAAGCTGGAGCTC
02181_compl_for	AAATCTAGACTGCTCACCGAGGTGGAGAG
02181_compl_rev	AAAGAATTCGGGTGCGCATGG
02181_exp_for	AAATCTAGAGAACCTATGTCCCTGAGTGACG
02181_exp_rev	AAAGAATTCCCATAAGGCAAGCGAGG
02181_460_for	AAAGGATCCGCTGCTCGACAACATCAC
02181_460_rev	AAAAAGCTTTCAAGTGGGTGGGACGGGAG
02181_460_aadef_for	GCCGCCGACGAGTTCGCGGCCCTG
02181_460_aadef_rev	CAGCCGGGAGGCGGTGTC
02181_EMSA_for	GCTTCGAGTACCGCCCG
02181_EMSA_rev	TCCGCACTGTCCGTCCC
Pnat_for	CAGGTGATCGTTACGGAGCGTAGAGAACCTATGTCCCTGAGTGACGATACTGG
Pnat_rev	CCAGTATCGTCACTCAGGGACATAGGTTCTCTACGCTCCGTAACGATCACCTG
PmutI_for	CAGGTGATCAAAAAAAAAAAAAAAAGAACCTATGTCCCTGAGTGACGATACTGG
PmutI_rev	CCAGTATCGTCACTCAGGGACATAGGTTCTTTTTTTTTTTTTTTGATCACCTG
PmutII_for	CAGGTGATCGTTACGGAGCGTAGAGAACCTAAAAAAAAAAAAAAAGATACTGG
PmutII_rev	CCAGTATCTTTTTTTTTTTTTTTAGGTTCTCTACGCTCCGTAACGATCACCTG
PmutIII_for	CAGGTGATCAAAAAAAAAAAAAAAAGAACCTAAAAAAAAAAAAAAAGATACTGG
PmutIII_rev	CCAGTATCTTTTTTTTTTTTTTTAGGTTCTTTTTTTTTTTTTTTGATCACCTG
02181_script_for	AAATCTAGACTCACCGAGGTGGAGAGC
02181_script_rev	AAAGGTACCCCCTGTCGGGACGATACAC
02181_check_pr_for	GCCACATCCTGGAACTG
02181_RT_rev	GGTCTTCTCGCTGTCCTC

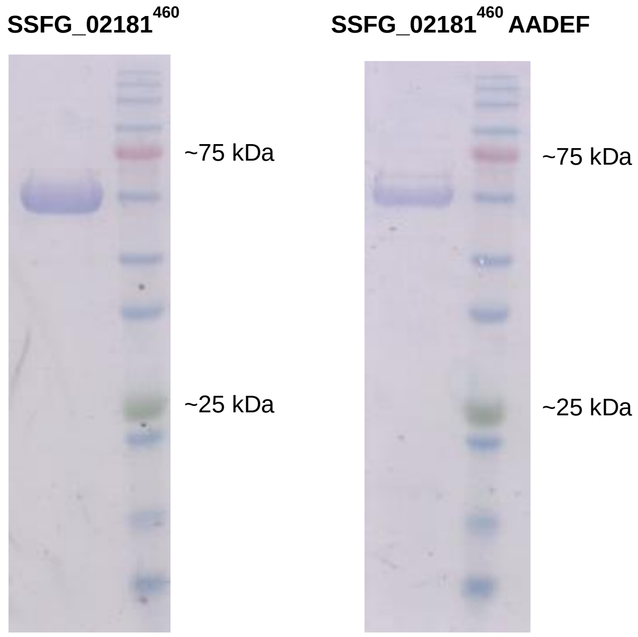

Figure S1. SSFG_02181^{460} and SSFG_02181^{460} AADEF proteins after Strep-tag purification and size exclusion chromatography.

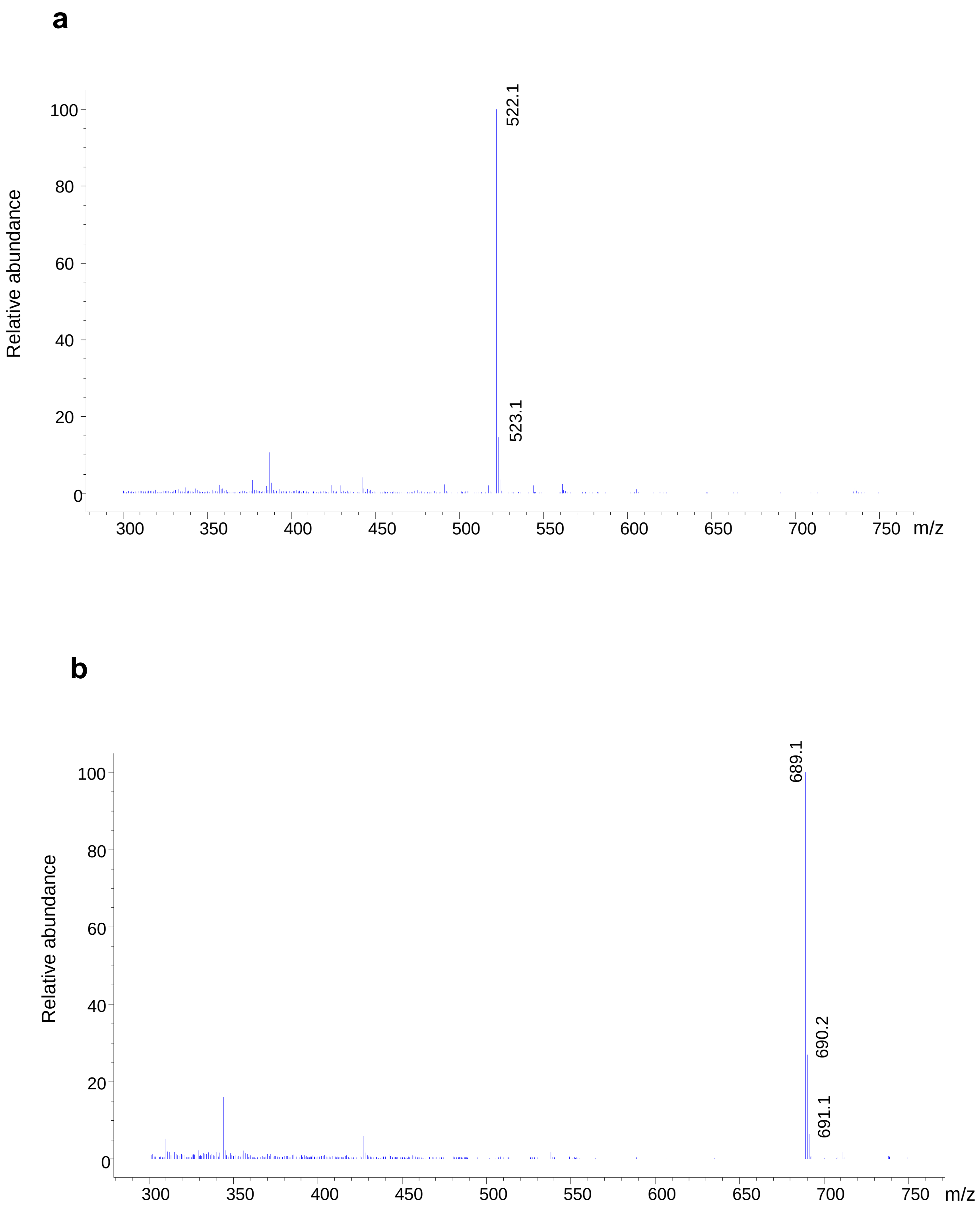

Figure S2. MS spectra of GTP and c-di-GMP detected after the DGC *in vitro* assay. **A**) $[M\text{-}H]^-$ ion of GTP (*m/z* 522.1). **B**) $[M\text{-}H]^-$ ion of c-di-GMP (*m/z* 689.1).

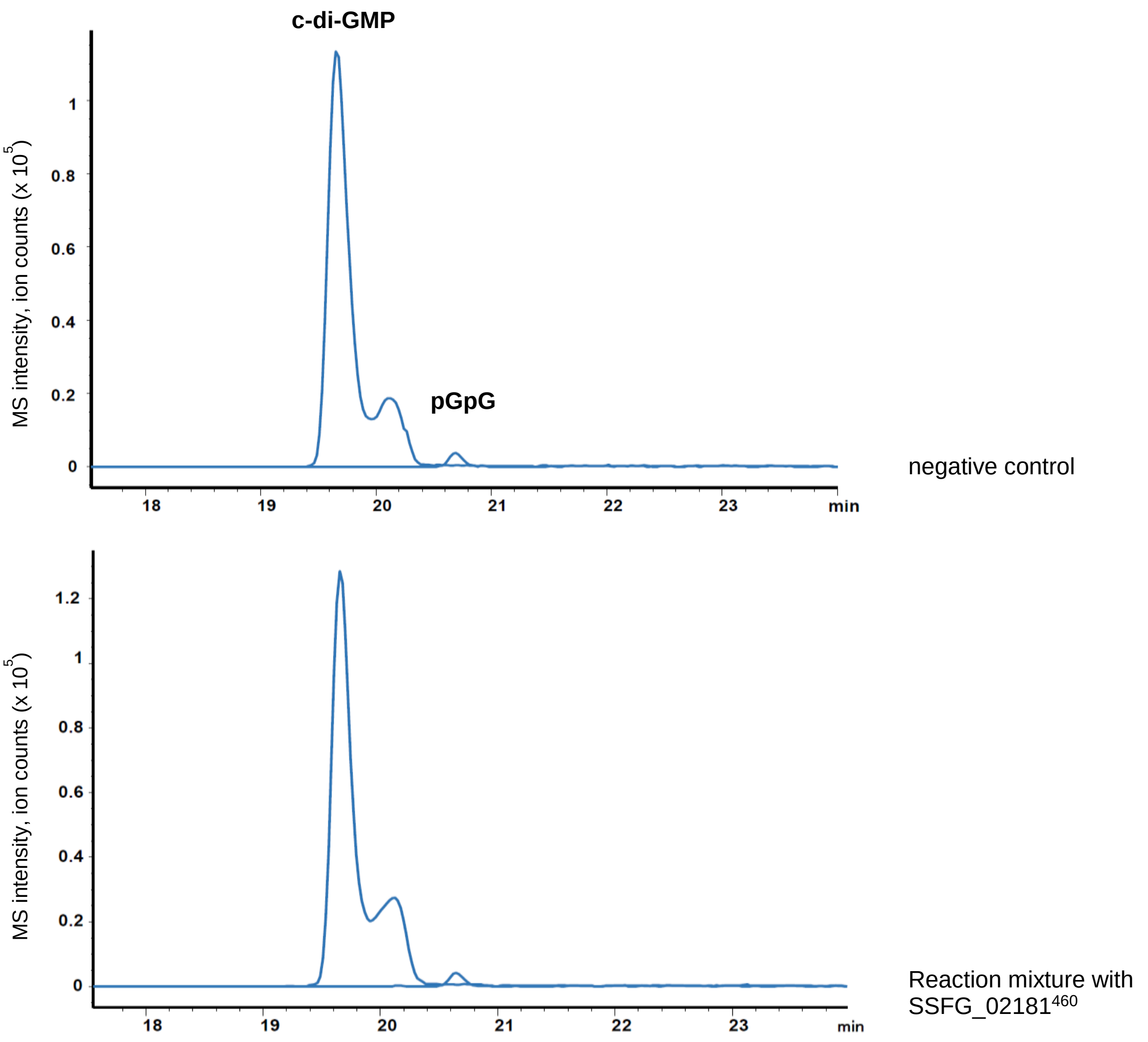

Figure S3. LC-MS chromatogram of *in vitro* phosphodiesterase assay. No conversion from c-di-GMP to pGpG was observed in the reaction mixture. EIC corresponding to the $[M\text{-}H]^-$ ion of pGpG (*m/z* 707.2) was detected in both control mixture and SSFG_02181^{460} reaction mixture as spontaneous degradation of c-di-GMP.

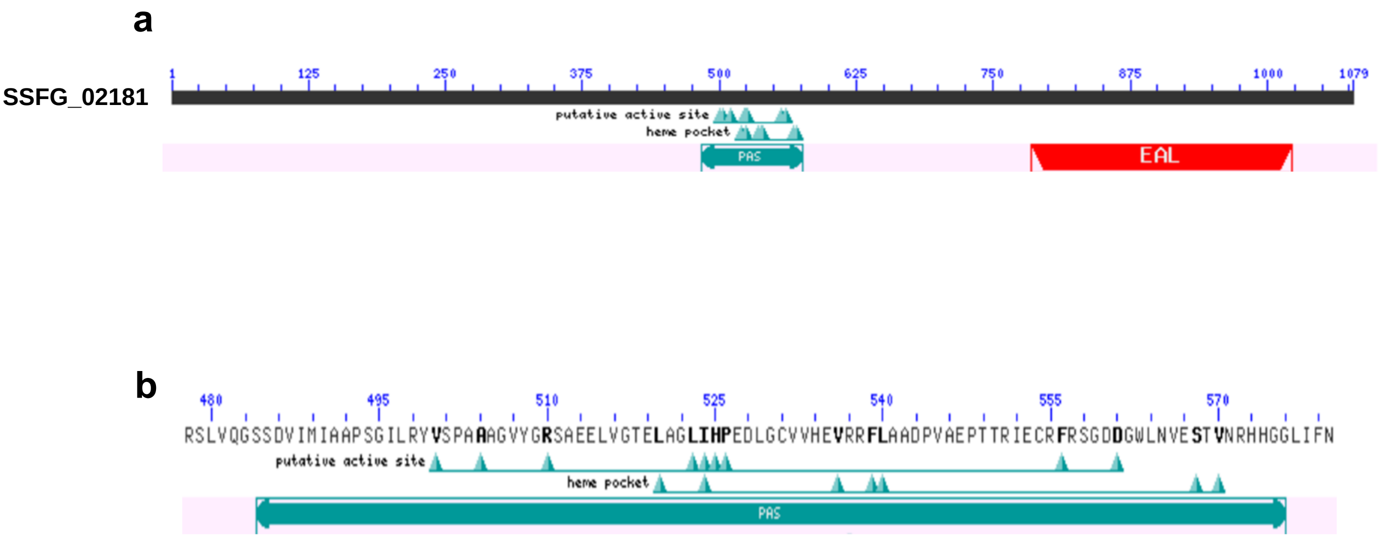

Figure S4. *In silico* identification of a putative heme pocket in SSFG_02181. **A**) BLAST analysis of the full SSFG_02181 revealed the presence of a putative heme pocket in its PAS sensor domain. **B**) Amino acid sequence of the PAS sensor domain. The predicted amino acids involved in the heme-binding are marked in bold.

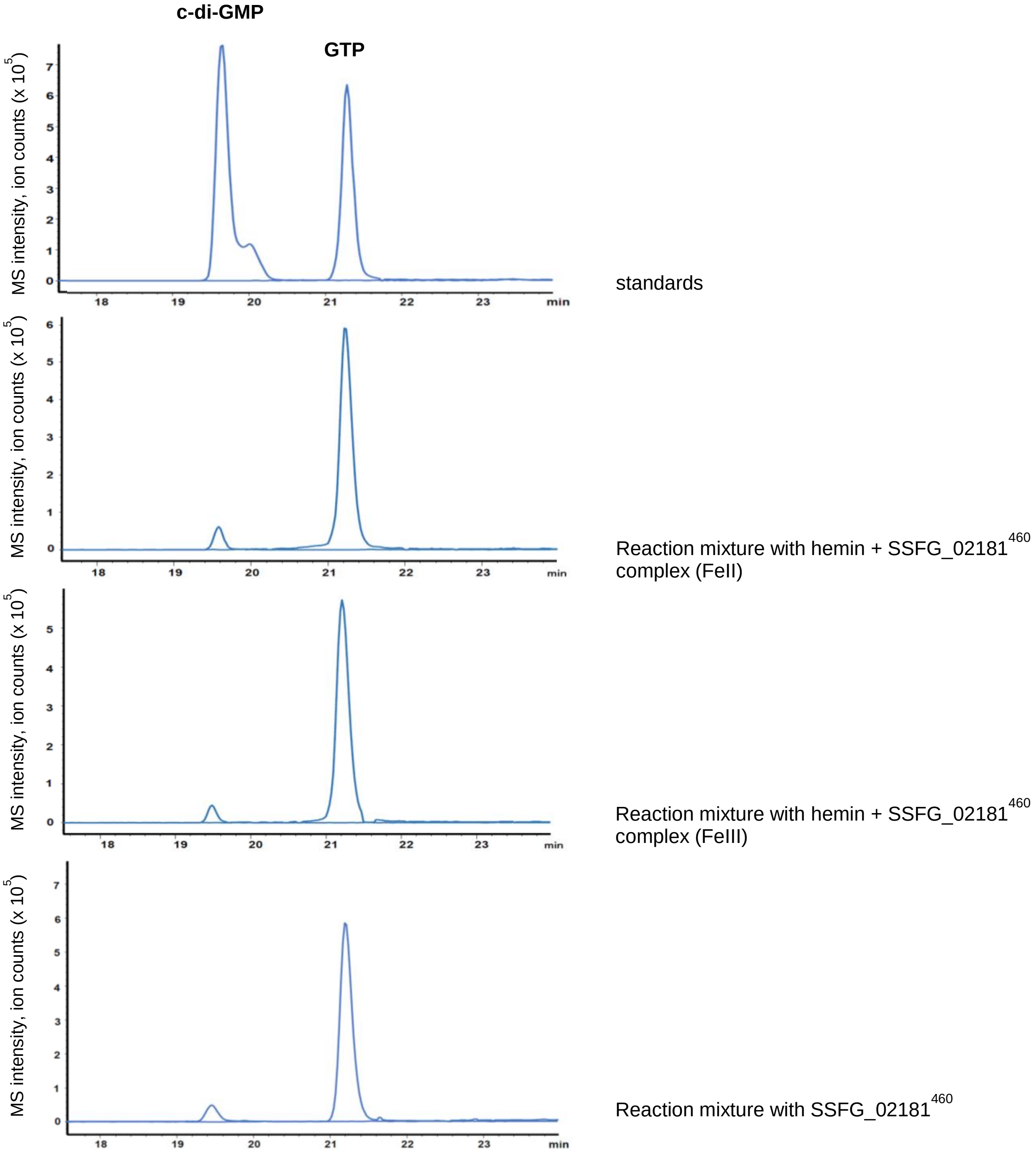

Figure S5. LC-MS detection of c-di-GMP synthesized by hemin-SSFG_02181^{460} complexes and SSFG_02181^{460} alone. EIC corresponding to the $[M\text{-}H]^-$ ion of c-di-GMP (*m/z* 689.1) was detected with no significant changes in the reaction mixtures of hemin-SSFG_02181^{460} (FeII) complex, hemin-SSFG_02181^{460} (FeIII) complex and SSFG_02181^{460} alone.

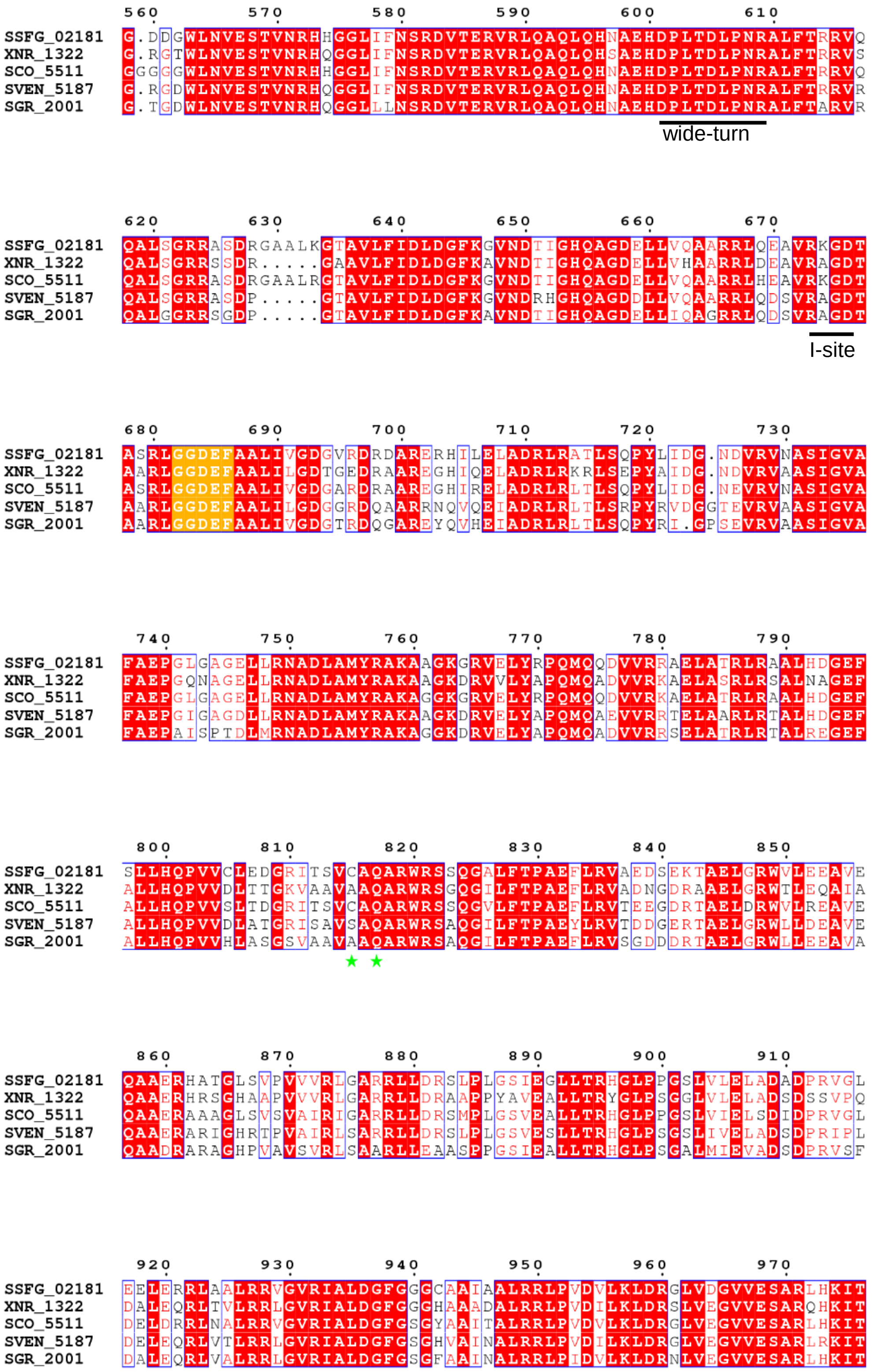

Figure S6. Multiple sequence alignment of SSFG_02181 with its homologs XNR_1322 (*S. albus*), SCO_5511 (*S. coelicolor*), SVEN_5187 (*S. venezuelae*) and SGR_2001 (*S. griseus*). The conservative GGDEF domain is highlighted in orange. A green star indicates amino acids from the degenerated EAL domain.

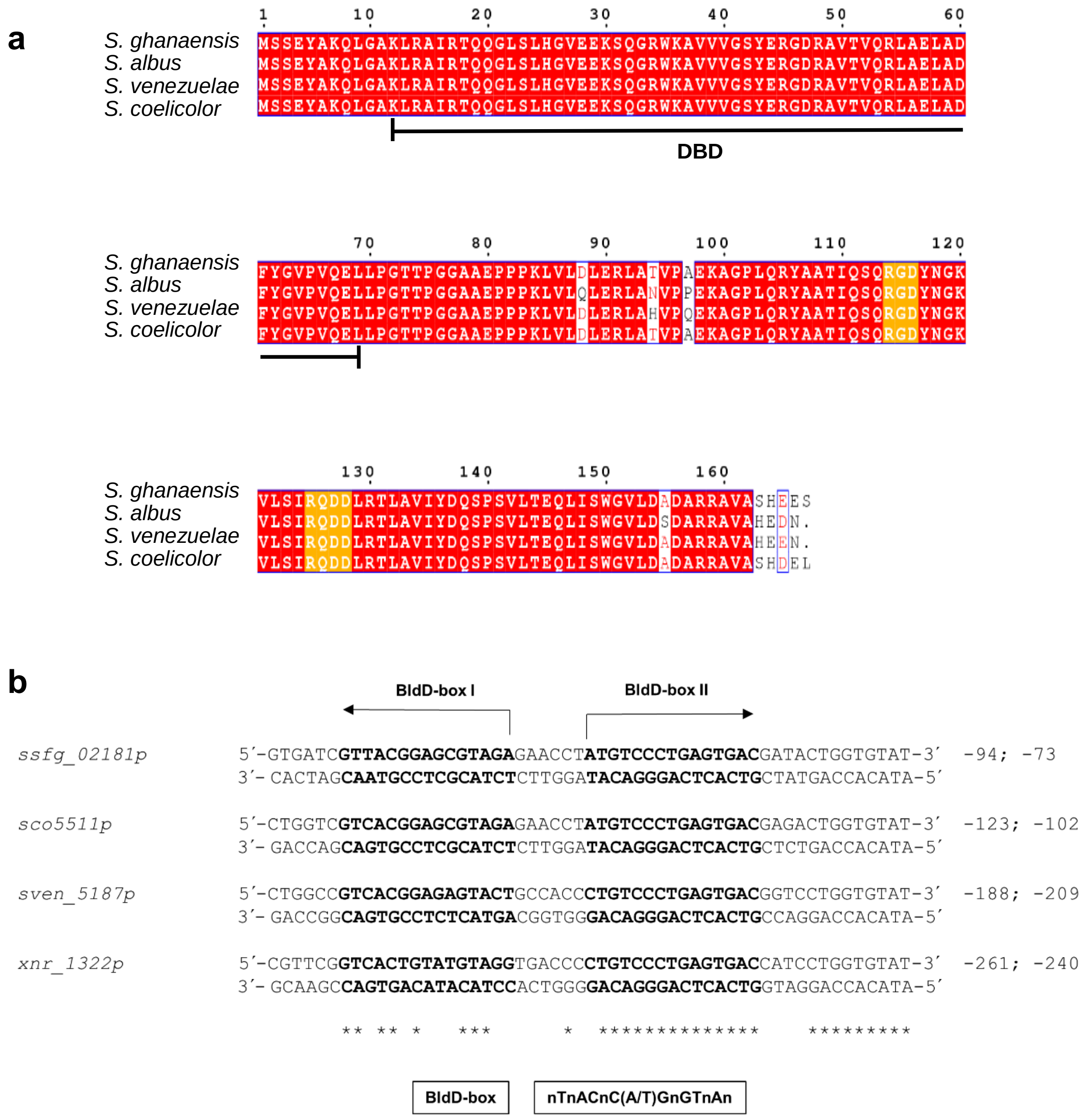

Figure S7. Multiple sequence alignment of BldD from *S. ghanaensis* with its orthologs and alignment of the putative BldD-boxes in *ssfg_02181* promoter and its orthologs. **A**) Multiple sequence alignment of BldD from *S. ghanaensis*, *S. coelicolor*, *S. venezuelae* and *S. albus*. A black bar indicates the DNA-binding domain (DBD), whereas the highly-conserved c-di-GMP binding motifs are highlighted in orange. **B**) The putative BldD-boxes from *ssfg_02181* promoter (*ssfg_02181p*), *sco5511* promoter (*sco5511p*), *sven_5187* promoter (*sven_5187p*) and *xnr_1322* promoter (*xnr_1322p*) are marked in bold. The asterisks indicate identical nucleotides and the numbers represent the distance from the putative start codon of the downstream gene.

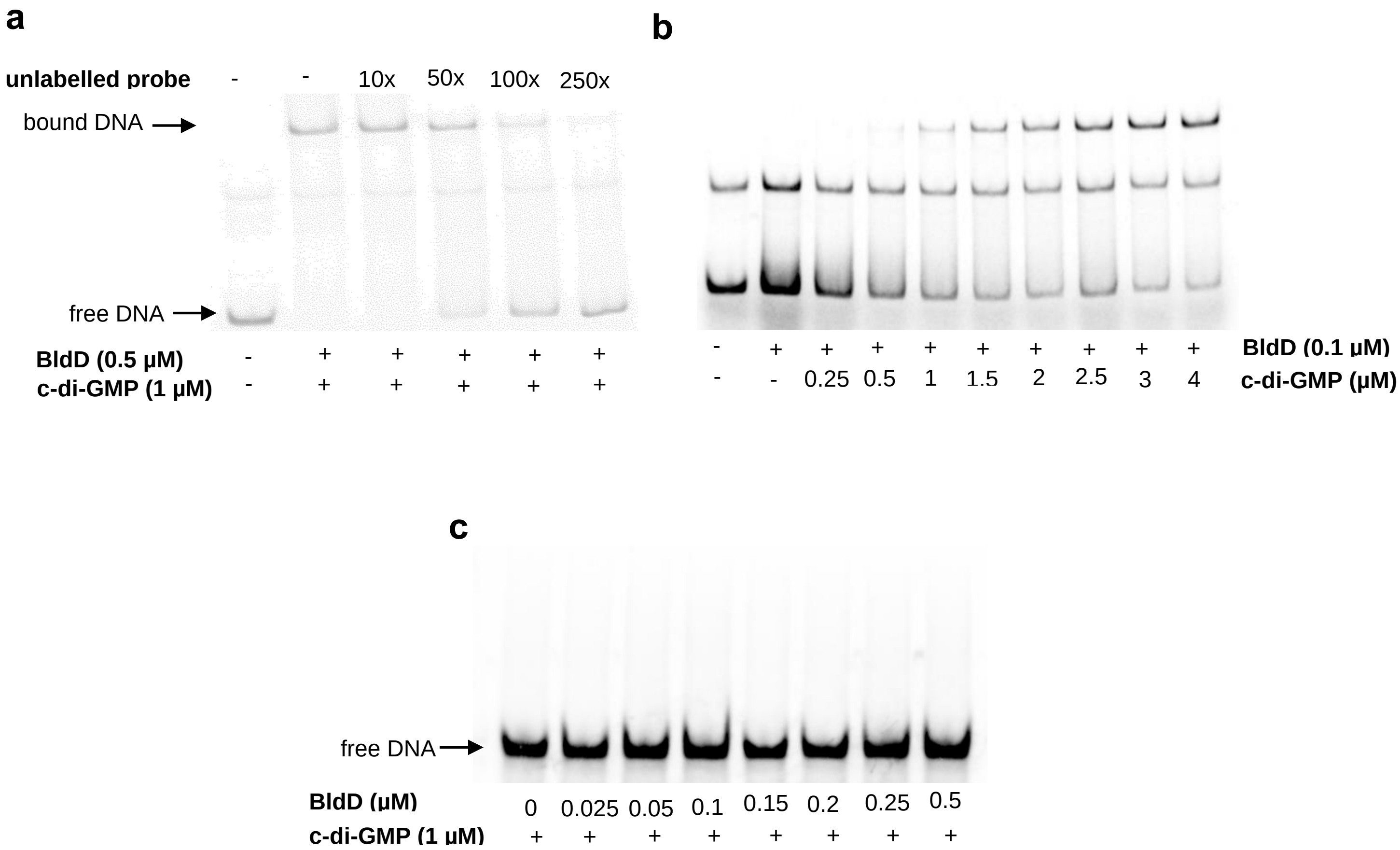

Figure S8. EMSA results of competition assay, c-di-GMP-dependent binding affinity evaluation and binding of BldD with a DNA hybrid carrying double mutation in BldD-boxes. **A**) EMSA competition assay of BldD with *ssfg_02181* promoter. **B**) Influence of c-di-GMP on BldD-*ssfg_02181* promoter binding affinity. **C**) EMSA analysis of BldD binding to a DNA fragment carrying mutations in both BldD-box I and BldD-box II.

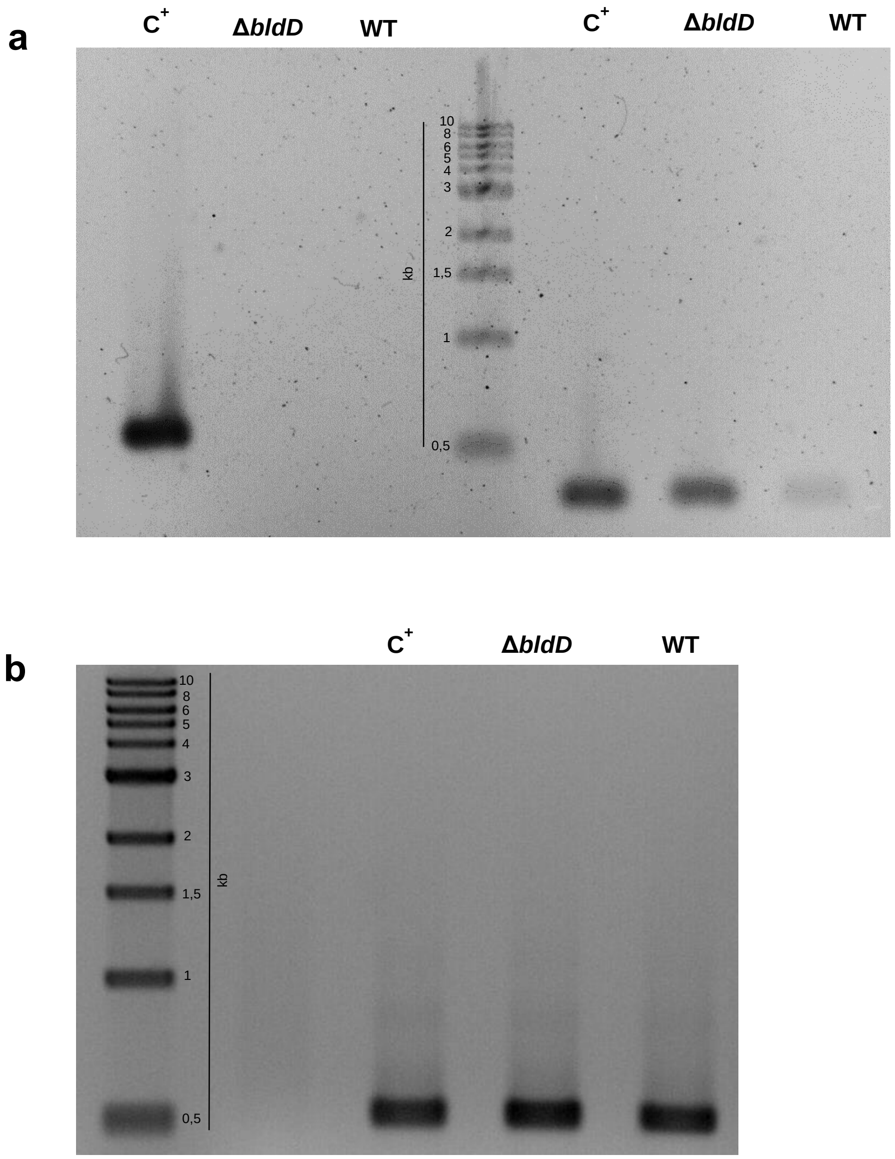

Figure S9. Unprocessed sqRT-PCR agarose gels. **A**) On the left is shown a negative control corresponding to the attempts to synthesize *hrdB* from RNA without pretreatment with Reverse transcriptase. On the right is shown the comparison of transcriptional profiles of *ssfg_02181* in the *S. ghananesis* wild-type (WT) and Δ*bldD* strains. C^+ (positive control) corresponds to the genomic DNA of *S. ghanaensis* WT strain. **B**) Synthesis of *hrdB* from the genomic DNA of *S. ghanaensis* WT strain (C^+), *S. ghanaensis* WT cDNA and Δ*bldD* cDNA strains.

REFERENCES

1. Makitrynskyy, R. *et al.* Secondary nucleotide messenger c-di-GMP exerts a global control on natural product biosynthesis in streptomycetes. *Nucleic Acids Res.*; 10.1093/nar/gkz1220 (2020).

2. Bentley, S. D. *et al.* Complete genome sequence of the model actinomycete *Streptomyces coelicolor* A3(2). *Nature* **417,** 141–147; 10.1038/417141a (2002).

3. Chater, K. F. & Wilde, L. C. *Streptomyces albus* G mutants defective in the SalGI restriction-modification system. *J. Gen. Microbiol.* **116,** 323–334; 10.1099/00221287-116-2-323 (1980).

4. Kieser, T. *Practical streptomyces genetics* (John Innes Foundation, Norwich, 2000).

5. Gust, B. *et al. in Advances in applied microbiology,* edited by A. I. Laskin, J. W. Bennett & G. M. Gadd (Elsevier, Estados Unidos, 2004), pp. 107–128.

6. Myronovskyi, M., Welle, E., Fedorenko, V. & Luzhetskyy, A. β-Glucuronidase as a Sensitive and Versatile Reporter in Actinomycetes. *Appl Env. Microbiol.* **77,** 5370–5383; 10.1128/AEM.00434-11 (2011).

7. Bierman, M. *et al.* Plasmid cloning vectors for the conjugal transfer of DNA from *Escherichia coli* to *Streptomyces* spp. *Gene* **116,** 43–49; 10.1016/0378-1119(92)90627-2 (1992).

8. Fedoryshyn, M., Welle, E., Bechthold, A. & Luzhetskyy, A. Functional expression of the Cre recombinase in actinomycetes. *Appl. Microbiol. Biotechnol.* **78,** 1065–1070; 10.1007/s00253-008-1382-9 (2008).

9. Herrmann, S. *et al.* Site-specific recombination strategies for engineering actinomycete genomes. *Appl. Env. Microbiol.* **78,** 1804–1812; 10.1128/AEM.06054-11 (2012).
